# The DUF59 Containing Protein SufT Is Involved in the Maturation of Iron-Sulfur (FeS) Proteins during Conditions of High FeS Cofactor Demand in *Staphylococcus aureus*

**DOI:** 10.1371/journal.pgen.1006233

**Published:** 2016-08-12

**Authors:** Ameya A. Mashruwala, Shiven Bhatt, Saroj Poudel, Eric S. Boyd, Jeffrey M. Boyd

**Affiliations:** 1 Department of Biochemistry and Microbiology, Rutgers University, New Brunswick, New Jersey, United States of America; 2 Department of Microbiology and Immunology, Montana State University, Bozeman, Montana, United States of America; 3 NASA Astrobiology Institute, Mountain View, California, United States of America; Virginia Tech, UNITED STATES

## Abstract

Proteins containing DUF59 domains have roles in iron-sulfur (FeS) cluster assembly and are widespread throughout Eukarya, Bacteria, and Archaea. However, the function(s) of this domain is unknown. *Staphylococcus aureus* SufT is composed solely of a DUF59 domain. We noted that *sufT* is often co-localized with *sufBC*, which encode for the Suf FeS cluster biosynthetic machinery. Phylogenetic analyses indicated that *sufT* was recruited to the *suf* operon, suggesting a role for SufT in FeS cluster assembly. A *S*. *aureus* Δ*sufT* mutant was defective in the assembly of FeS proteins. The DUF59 protein Rv1466 from *Mycobacterium tuberculosis* partially corrected the phenotypes of a Δ*sufT* mutant, consistent with a widespread role for DUF59 in FeS protein maturation. SufT was dispensable for FeS protein maturation during conditions that imposed a low cellular demand for FeS cluster assembly. In contrast, the role of SufT was maximal during conditions imposing a high demand for FeS cluster assembly. SufT was not involved in the repair of FeS clusters damaged by reactive oxygen species or in the physical protection of FeS clusters from oxidants. Nfu is a FeS cluster carrier and *nfu* displayed synergy with *sufT*. Furthermore, introduction of *nfu* upon a multicopy plasmid partially corrected the phenotypes of the Δ*sufT* mutant. Biofilm formation and exoprotein production are critical for *S*. *aureus* pathogenesis and vancomycin is a drug of last-resort to treat staphylococcal infections. Defective FeS protein maturation resulted in increased biofilm formation, decreased production of exoproteins, increased resistance to vancomycin, and the appearance of phenotypes consistent with vancomycin-intermediate resistant *S*. *aureus*. We propose that SufT, and by extension the DUF59 domain, is an accessory factor that functions in the maturation of FeS proteins. In *S*. *aureus*, the involvement of SufT is maximal during conditions of high demand for FeS proteins.

## Author Summary

Iron-sulfur (FeS) clusters are inorganic cofactors that are used for diverse cellular processes including cellular respiration, DNA replication and repair, antibiotic resistance, and dinitrogen fixation. A failure to properly assemble FeS clusters in proteins results in widespread metabolic disorders, metabolic paralysis, and oftentimes cell death. Therefore, the biosynthesis of FeS clusters is essential for nearly all organisms. Proteins containing DUF59 domains are widespread in Eukarya, Bacteria, and Archaea. Proteins containing DUF59 domains have roles in FeS cluster assembly, but the function(s) of the DUF59 domain is unknown. Moreover, the function(s) of proteins containing DUF59 domains are largely unknown. *Staphylococcus aureus* SufT is composed solely of a DUF59 domain, which provides a unique opportunity to examine the role(s) of this domain in cellular physiology. In this report we show SufT to be an accessory factor utilized in FeS cluster assembly during conditions imposing a high-demand for FeS proteins. We also show that deficiencies in the maturation of FeS proteins result in alterations in the ability of *S*. *aureus*, an epidemic human pathogen, to form biofilms, produce exoproteins, and resist antibiotic stress.

## Introduction

Iron (Fe) is an essential nutrient for nearly all organisms. Fe is acquired from the environment and is transported into cells using specific uptake systems. Studies have shown that ~80% of the intracellular Fe is located in inorganic cofactors, called iron-sulfur (FeS) clusters, and heme in a respiring microorganism [1].

The metabolisms of most organisms are highly reliant on FeS cluster chemistry and a failure to properly assemble FeS clusters in proteins can result in widespread metabolic disorders, metabolic paralysis, and cell death [2,3,4]. FeS proteins function in diverse metabolic processes including environmental sensing[5], carbon transformations [6], DNA repair and replication [7,8], RNA metabolism [9], protein synthesis [10], nucleotide, vitamin, and cofactor synthesis [11,12,13], and cellular respiration [14,15,16]. FeS clusters are typically found in proteins as [Fe_2_S_2_] or [Fe_4_S_4_] clusters, but the use of complex FeS clusters has evolved for processes such as dinitrogen [17], carbon monoxide [18], and hydrogen metabolism [19].

Iron and sulfur (S) ions are often toxic to cells resulting in the evolution of tightly controlled mechanisms to synthesize FeS clusters from their monoatomic precursors [20,21]. Three FeS cluster biosynthetic systems (Nif, Suf, and Isc) have been described in Bacteria and Archaea for the synthesis of [Fe_2_S_2_] and [Fe_4_S_4_] clusters [22,23,24]. Bioinformatic analyses suggest that the Suf system is the most prevalent machinery in Bacteria and Archaea and perhaps the most ancient [25].

The Suf, Nif, and Isc systems utilize a common strategy to synthesize FeS clusters. First, sulfur is mobilized from free cysteine (typically), using a cysteine desulfurase enzyme and subsequently transferred to either a sulfur carrier molecule (SufU or SufE) or directly to the synthesis machinery [24,26,27]. Monoatomic iron and sulfur, along with electrons, are combined upon a molecular scaffolding protein (SufBD in *S*. *aureus*) to form an FeS cluster [28]. The FeS cluster can be transferred directly from the scaffold to a target apo-protein or it can be transferred to a carrier molecule that subsequently traffics the cluster to a target apo-protein and facilitates maturation of the holo-protein [29]. Nfu and SufA serve as FeS cluster carriers in *Staphylococcus aureus* [4,30]. Nfu is necessary for virulence in models of infection [4,31]

Most studies on bacterial FeS cluster assembly have been conducted using *Escherichia coli* and *Azotobacter vinelandii*. *E*. *coli* encodes for both the Suf and Isc systems [22] whereas *A*. *vinelandii* encodes for the Isc and Nif systems [32]. In contrast, few studies have been conducted on FeS cluster assembly in gram-positive bacteria such as *Bacillus subtilis* or *S*. *aureus*, which encode for only the Suf system [4,27]. Recent findings suggest that SufCDSUB are essential for *S*. *aureus* viability, confirming that Suf is the sole FeS cluster biosynthetic machinery used under laboratory growth conditions [4,33,34].

Dioxygen can accept electrons from cellular factors resulting in the spontaneous generation of reactive oxygen species (ROS) such as hydrogen peroxide (H_2_O_2_) and superoxide [35,36,37]. FeS clusters are among the primary cellular targets of H_2_O_2_ and superoxide [38,39]. ROS readily oxidize solvent exposed [Fe_4_S_4_]^2+^ cofactors of enzymes such as aconitase (AcnA) [38,39]. Oxidation results in conversion to an inactive [Fe_3_S_4_]^1+^ cluster that can be repaired back to the active [Fe_4_S_4_]^2+^ state using Fe^2+^ and an electron [40]. Studies have implicated roles for cysteine desulfurase (IscS) and the putative Fe donors CyaY, YtfE, and YggX in the repair of oxidized clusters [40,41,42]. Cells also employ mechanisms to physically protect FeS clusters. The Shethna protein shields the FeS cofactor of dinitrogen reductase from dioxygen exposure [43]. Alternatively, protein domains can be situated in a manner that prevents oxidants from interacting with the FeS cluster. The pyruvate:ferredoxin oxidoreductase (PFOR) from *Desulfovibrio africanus* was found to have greater stability in the presence of dioxygen, relative to alternate PFOR enzymes, due to the presence of a domain that prevents the interaction of oxidants with its [Fe_4_S_4_]^2+^ cluster [44].

We have identified an open reading frame (ORF) in *S*. *aureus* that is often associated with the *suf* operon in a number of bacterial and archaeal genomes. The ORF (SAUSA300_0875) encodes for a protein composed solely of a DUF59 domain and is annotated as SufT since it is often found in operons with a cysteine desulfurase (i.e. SufS) [45]. In eukaryotic cells, the CIA2 (also identified as Fam96a/b or AE7) FeS cluster assembly factor(s) contain a DUF59 domain [46,47]. CIA2a and CIA2b act downstream of the cytosolic iron-sulfur assembly (CIA) machinery and are required for the maturation of FeS cluster proteins. A DUF59 domain is also present in the *Arabidopsis thaliana* chloroplast FeS cluster carrier, HCF101, which is required for photosystem I maturation [48].

*S*. *aureus* is a leading cause of human infectious disease related morbidity and mortality worldwide. *S*. *aureus* forms surface associated communities referred to as biofilms that are critical for *S*. *aureus* pathogenesis and biofilm associated cells serve as the etiologic agents of recurrent staphylococcal infections (reviewed here [49]). *S*. *aureus* also secretes a variety of toxins and enzymes into its extracellular milleu that are critical for biofilm formation, host colonization, nutrient acquisition and survival in the human host (reviewed here [50]). About 60% of the secretome consists of peptide toxins (phenol soluble modulins (PSM's), which have multiple key roles in pathogenesis [51,52].

Since the 1990s the proportion of infections caused by community-associated methicillin resistant *S*. *aureu*s (CA-MRSA) has been steadily increasing and has now reached near epidemic levels [53]. Vancomycin is a glycopeptide antibiotic that has traditionally been regarded as a last-resort drug for the treatment of MRSA infections [54]. Strains have recently emerged that display intermediate (vancomycin intermediate-resistant *S*. *aureus*; VISA) or high (vancomycin resistant *S*. *aureus*; VRSA) levels of resistance towards vancomycin [54,55]. Among the characteristics of VISA strains are decreased activity of peptidoglycan hydrolases and alterations in their cell wall that results in increased resistance to the lytic enzyme lysostaphin [55].

*S*. *aureus* provides an excellent model to assess the role of the DUF59 domain (SufT) in cellular physiology. In this report we present phylogenetic analyses indicating a widespread distribution for SufT and conservation of SufT homologs in bacterial and archaeal taxa that utilize the Suf system. These analyses also suggest that *sufT* was recruited to the neighborhood of *sufBC* over evolutionary time and for the most part retained. The bioinformatic analyses led us to hypothesize that SufT has a role in the maturation of FeS proteins. Results demonstrate an involvement of SufT in the maturation of FeS proteins during conditions imposing a high demand for FeS proteins. Moreover, epistasis studies show that the *nfu* and *sufT* mutations display synergy and the introduction of *nfu* in multicopy partially corrects the phenotypes of a *sufT* mutant. Deficiencies in the maturation of FeS proteins also result in increased biofilm formation, decreased exoprotein production, and the appearance of phenotypes consistent with vancomycin-intermediate resistant *S*. *aureus* (VISA). We propose that SufT functions as an auxiliary factor for the maturation of FeS proteins with maximum usage during conditions of high FeS cofactor demand.

## Results

### Recruitment of *sufT* to the *suf* operon suggests that it functions in FeS cluster assembly

Of the 1669 complete genome sequences available as of October 2011 and compiled as part of our previously published work on the evolution of Suf [25], 1092 (65.4% of total) encoded for SufBC. Among these genomes, 761 (69.7% of total) encoded for SufT. Of the 1669 genomes, 68 genomes contained *sufT*, but not *sufBC*. Five genomes contained *sufT*, but not *sufB*, *iscU*, or *nifU*, which encode for FeS cluster scaffolding molecules. These genomes were all from lactobacilli and the *sufT* homologues are in apparent operons with the genes encoding for either anaerobic ribonucleoside-triphosphate activating enzyme or serine dehydratase, which are FeS cluster-requiring enzymes [11,56].

**Fig 1:**
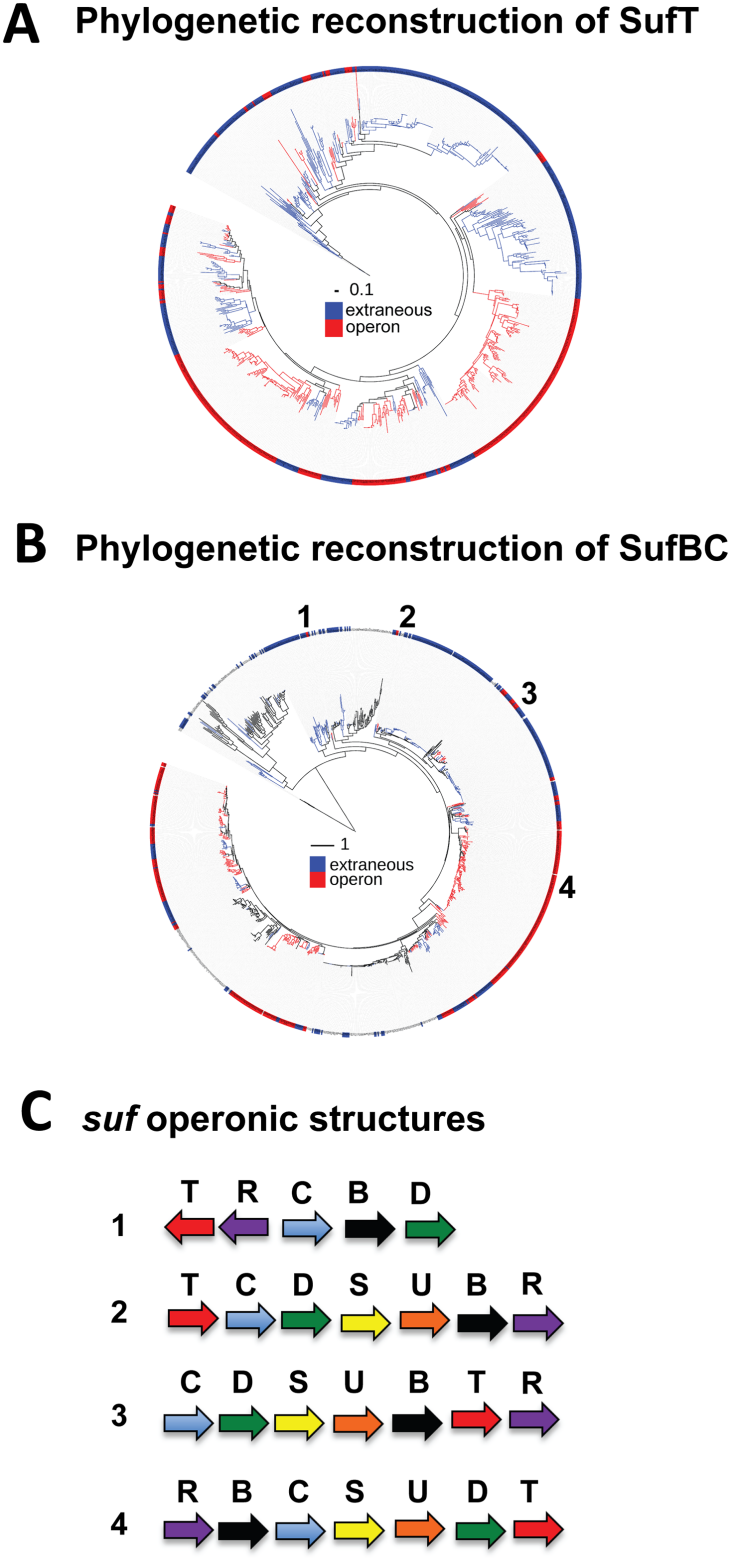
Phylogenetic analyses of *sufT* containing operons. Panel A: Maximum likelihood phylogenetic reconstruction of 761 SufT homologs compiled from 1092 genome sequences that also encoded for SufBC. SufT lineages were color coded red if they were associated with *sufBC* in the genome (within four open reading frames of *sufBC*) or blue if they were encoded in another part of the genome. The tree is unrooted and the tree was constructed as reported in the materials and methods. Panel B: Maximum likelihood phylogenetic reconstruction of a concatenation of 1094 SufBC homologs. SufBC lineages were color-coded red if *sufT* was within four ORFs of *sufBC* in the genome and were color-coded blue if *sufT* was encoded in another part of the genome. The SufBC tree is rooted and was constructed as previously described [25]. Panel C: Select *suf* operonic structures from the data displayed in Panel B. The *suf* operons from: 1. *Thermoplasma acidophilum* DSM 1728, 2. *Alicyclobacillus acidocaldarius* subsp. *acidocaldarius* DSM 446, 3. *Lactobacillus reuteri* SD2112, and 4. *Mycobacterium tuberculosis* H37Rv are shown and are mapped on the SufBC phylogeny in Panel B.

Among the 761 genomes that encoded for *sufT* and *sufBC*, 374 of the *sufT* homologs were localized with *sufBC* (*suf* operon associated) and 387 *sufT* homologs were not associated with *sufBC* (non-*suf* operon associated). Maximum likelihood phylogenetic reconstructions of SufT (unrooted) and SufBC (rooted), followed by overlays of *suf*-operon associated and non-*suf* operon associated *sufT*, indicate that *sufT* has been recruited to and lost from the *suf* operon multiple times during its evolutionary history (Fig 1). However, the overall trend appears to be retainment once *sufT* was recruited to the *suf* operon. Mapping of the association of *sufT* with the *suf* operon on the SufBC tree indicates that *sufT* was not associated with the *suf* operon early during the evolution of taxa that used the Suf FeS cluster biosynthetic system and that it was recruited to the operon recently in its evolutionary history. Each SufT homolog identified contained a conserved cysteine residue, which was previously shown to be hyper-reactive [57], but described FeS cluster-binding motifs were not recognized.

**Fig 2:**
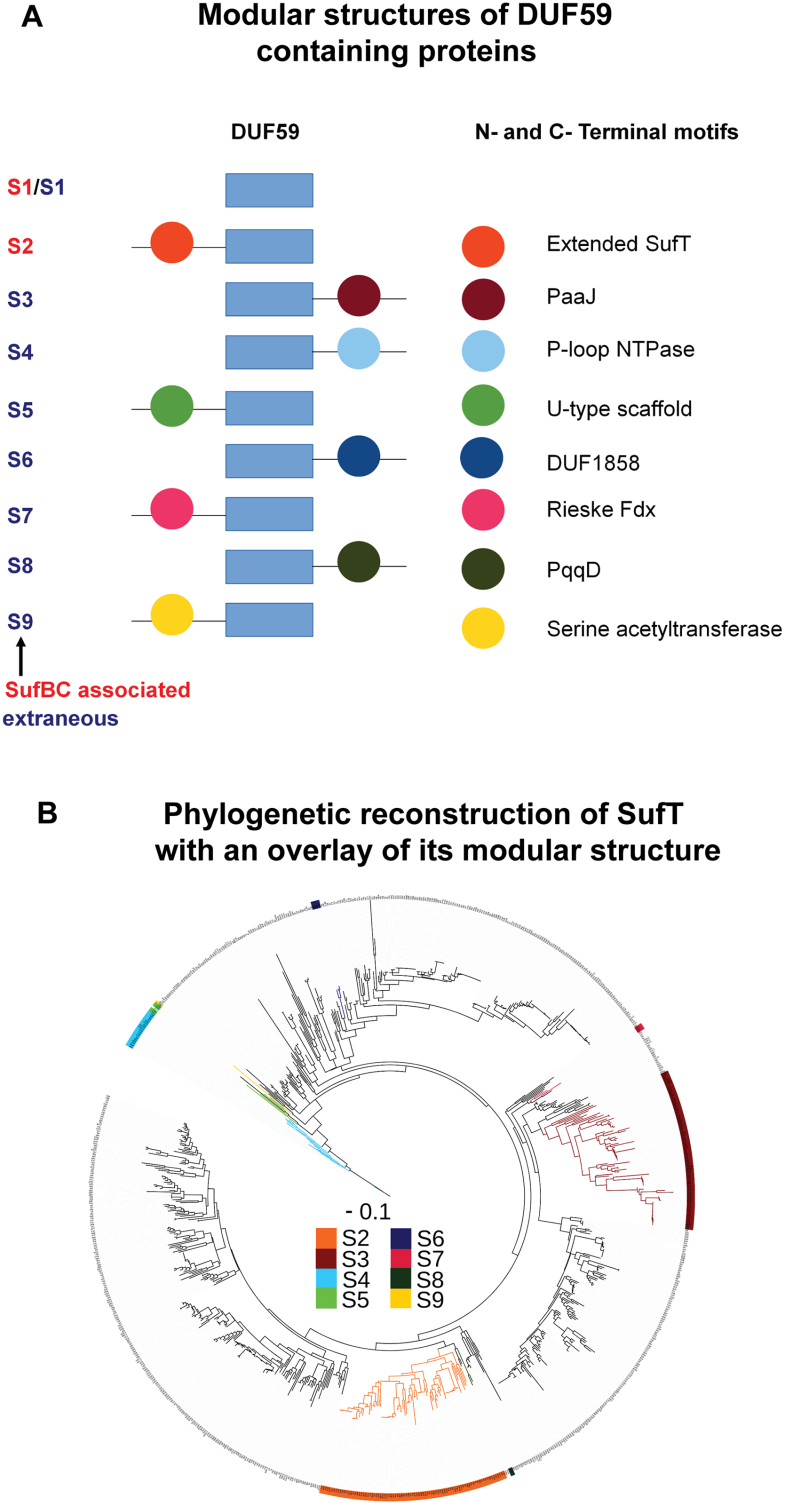
Modular structures of SufT homologs containing DUF59 domains. Panel A: Modular structures of DUF59 containing proteins as determined with sequence alignments and BLASTp against the conserved domain database [103]. The nine modular structures, referred to as S2 to S9, are depicted with red module labels corresponding to *sufT* that are within four ORFs of *sufBC* in the genome, or blue module labels corresponding to *sufT* encoded elsewhere in the genome. N- and C-terminal motifs are indicated. The *S*. *aureus* SufT is a representative member of the S1 structure. Panel B: Maximum likelihood phylogenetic reconstruction of SufT with an overlay of S2 to S9 modular structures. For simplicity, the most prevalent configuration (S1) was not mapped.

Of the total (n = 761) identified SufT homologs, the predominant structure contained only the DUF59 domain (S1 architecture; ex. *S*. *aureus* SufT), but 198 encoded for additional N- and C-terminal motifs represented by nine primary modular structures (Fig 2A). The most prevalent modular structure was the S2 architecture (n = 88), with a N-terminal motif that did not display homology to previously described domains. SufT within the S5 architecture (n = 5) contained a N-terminal domain with homology to U-type FeS cluster scaffolds while SufT within the S7 architecture (n = 3) harbored a N-terminal domain with homology to Rieske iron-oxygenase ferredoxins. Finally, SufT within the S9 architecture (n = 1) contained a N-terminal domain with homology to serine acetyltransferases (CysE). Characterization of the C-terminal motifs also revealed variation that was represented in four unique modular structures. These were characterized as SufT with C-terminal domains that have homology to PaaJ or acetyl-CoA acetyltransferase domains (S3 architecture, n = 75), P-loop NTPase domains (S4 architecture n = 20), DUF1858 domains (S6 architecture, n = 4) and co-enzyme pyrroloquinoline quinone synthesis protein D (PqqD) domains (S8 architecture, n = 2). The S2-S9 architectures were mapped on the phylogenetic reconstruction of core DUF59 (N- and C-terminal motifs were pruned from alignment block) in order to determine if the modules are randomly distributed over the tree or if they are phylogenetically clustered. The overall pattern of clustering of the modular structures on the tree (Fig 2B) indicates that once these modules were fused to an ancestor of a given DUF59 containing protein, they were largely retained. This suggests that the N- and C-terminal motifs, and presumably their functionalities, are under strong selective pressure.

### A Δ*sufT* mutant has decreased activity of the FeS cluster-requiring enzyme AcnA

We created and characterized a *S*. *aureus* Δ*sufT* mutant to test whether SufT has a role in the maturation of FeS proteins.

**Fig 3:**
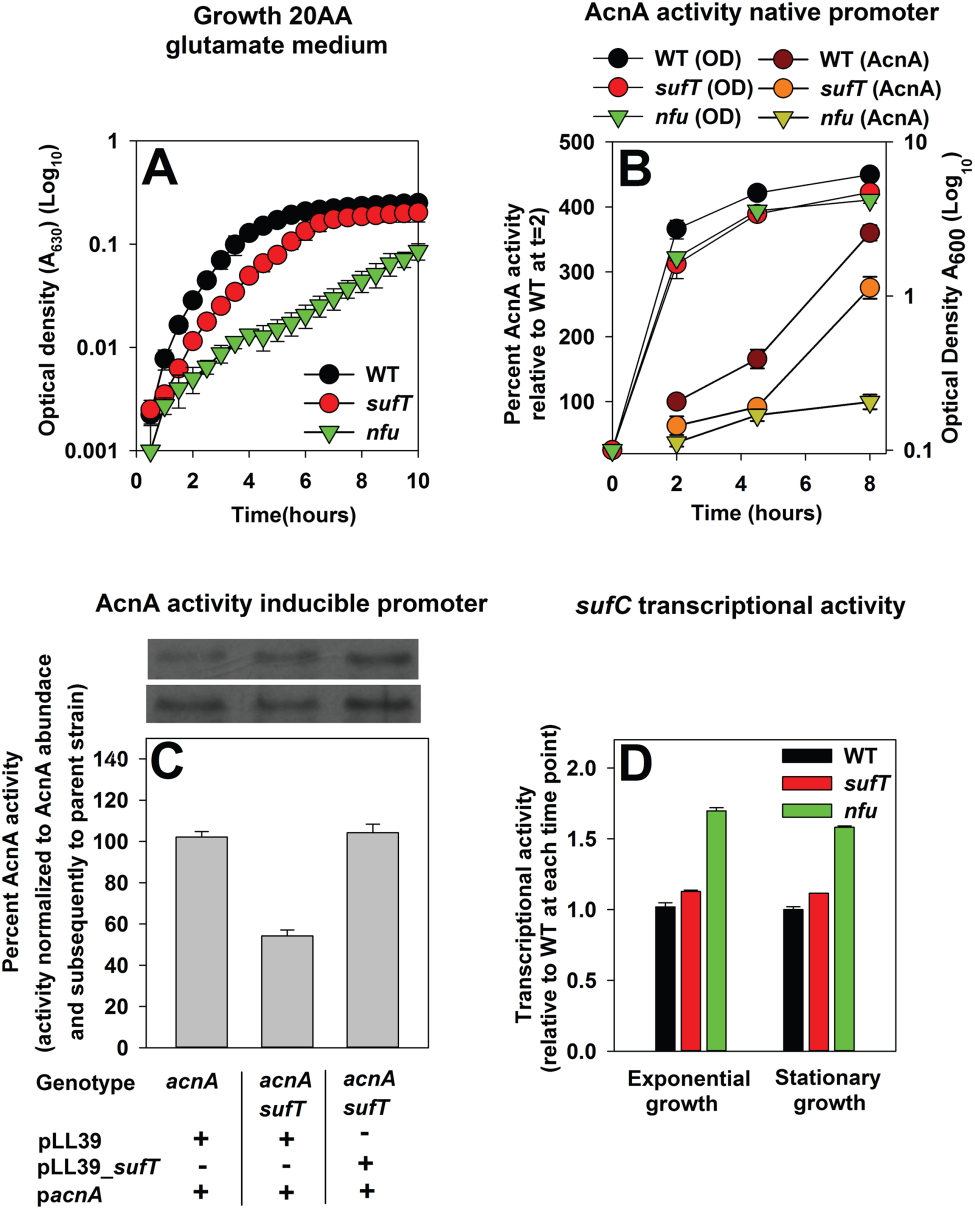
Analyses of Aconitase function in a Δ*sufT* strain. Panel A: *S*. *aureus* Δ*nfu* and Δ*sufT* mutant strains are defective for growth with glutamate as a carbon source. Growth traces of the WT (JMB1100), Δ*sufT* (JMB1146), and Δ*nfu* (JMB1165) strains in defined minimal medium containing the canonical 20 amino acids and glutamate as a carbon source (20AA glutamate medium). Panel B: Aconitase (AcnA) activity is decreased in strains lacking SufT or Nfu. Culture optical densities, as well as AcnA activities were assessed for the WT (JMB1100), Δ*sufT* (JMB1146), and Δ*nfu* (JMB1165) strains over the course of aerobic growth. Panel C: AcnA activity is decreased in a *sufT* mutant independent of *acnA* transcription and AcnA abundance. AcnA activity was assessed from the *acnA*::*TN* (JMB4432), *acnA*::*TN* Δ*sufT* (JMB4374), and the genetically complemented *acnA*::*TN* Δ*sufT sufT*^+^ (JMB4373) strains. All strains contained the p*acnA* plasmid, which contains *acnA* under the transcriptional control of a xylose inducible promoter. Top: Western blot analyses of the AcnA_FLAG displaying AcnA protein abundance in each strain, determined in duplicate. Panel D: Transcriptional activity of *sufC* is not decreased in Δ*nfu* and Δ*sufT* mutant strains. Transcriptional activity of *sufC* was assessed in the WT (JMB1100), Δ*sufT* (JMB1146), and Δ*nfu* (JMB1165) strains. The data represent the average of four (Panel A) or three (Panel B, C, and D) biological replicates and error bars represent standard deviations. Error bars are shown in all figures but may not be visible where error is low.

A *S*. *aureus* Δ*acnA* strain is defective in utilizing glutamate as a source of carbon (S1A Fig) [58,59]. Nfu has a role in the maturation of AcnA in *S*. *aureus* [4]. The Δ*nfu* and Δ*sufT* strains displayed growth defects in chemically defined media supplemented with glutamate as a carbon source (hereafter 20AA glutamate medium) (Fig 3A), but the defect of the Δ*sufT* strain was less severe than that of the Δ*nfu* strain. The WT, Δ*nfu*, and Δ*sufT* strains had similar growth profiles in defined medium containing glucose as a carbon source (hereafter 20 AA glucose medium) (S1B Fig).

AcnA activity was assessed in the WT, Δ*sufT*, and Δ*nfu* strains across growth. AcnA activity was decreased in strains lacking Nfu or SufT (Fig 3B). The decreased AcnA activity in the Δ*sufT* strain could arise due to one of four scenarios: 1) decreased transcription of *acnA*, 2) decreased abundance of AcnA, 3) decreased occupancy of the [Fe_4_S_4_] cluster upon AcnA due to the decreased transcription of genes encoding FeS cluster biogenesis factors, or 4) decreased cluster occupancy upon AcnA due to the absence of SufT.

Transcriptional activity of *acnA* was increased in the Δ*sufT* strain (S2 Fig). This suggested that decreased AcnA activity in the Δ*sufT* strain was not the result of altered *acnA* transcription (S2 Fig). We constructed *acnA*::*TN* strains containing a plasmid with a *acnA_FLAG* allele under the transcriptional control of a xylose inducible promoter (p*acnA*). Introduction of p*acnA* allows for the control of *acnA* transcription and the simultaneous determination of AcnA_FLAG abundance. The *acnA*::*TN* Δ*sufT* strain was genetically complemented by re-introduction of the *sufT* allele at a secondary chromosomal location (*sufT*+). AcnA activity and AcnA abundance was assessed in the *acnA*::*TN*, *acnA*::*TN* Δ*sufT*, and *acnA*::*TN* Δ*sufT sufT*+ strains containing p*acnA*. AcnA activity was ~2-fold lower in the *acnA*::*TN* Δ*sufT* strain compared to the *acnA*::*TN* when activity was normalized to AcnA abundance in the same cell-free lysates (Fig 3C). This phenotype was genetically complemented.

Suf is encoded by the *sufCDSUB* operon in *S*. *aureus*. The transcriptional activity of *sufC* was increased (~2-fold) in the Δ*nfu* strain and mildly, but consistently, increased in the Δ*sufT* strain (Fig 3D). Similar results were obtained in exponential and stationary growth. From Fig 3 we concluded that the absence of SufT results in decreased occupancy of the [Fe_4_S_4_] cofactor upon AcnA.

### A *sufT* mutant has a general defect in FeS cluster assembly

**Fig 4:**
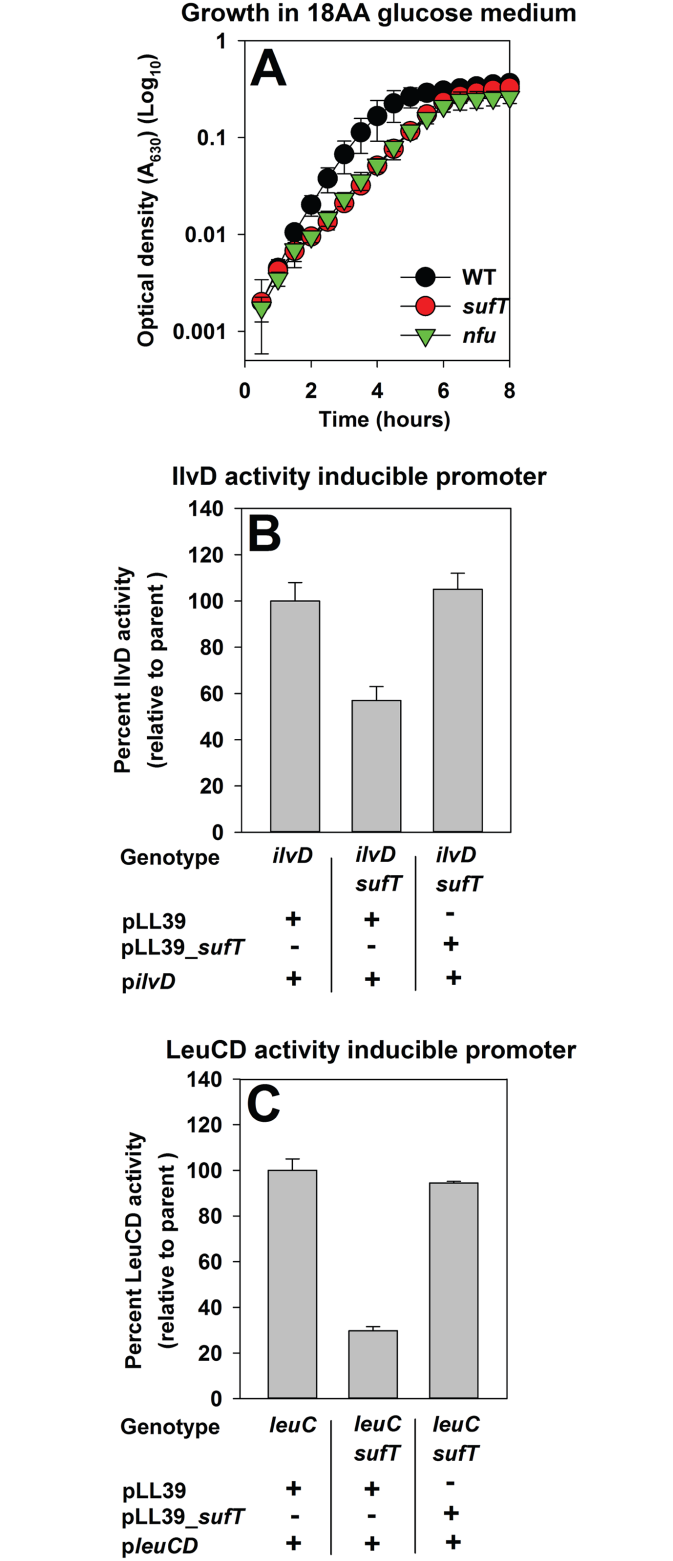
Necessity of SufT for LeuCD and IlvD function. Panel A: Strains lacking either SufT or Nfu are defective for growth in a medium lacking the amino acids leucine and isoleucine. Growth traces of the WT (JMB1100), Δ*sufT* (JMB1146), and Δ*nfu* (JMB1165) strains in defined minimal medium containing glucose as a carbon source and the canonical amino acids except leucine and isoleucine (18AA glucose medium). Panel B and C: IlvD and LeuCD activities are decreased in a strain lacking SufT, which is independent of *ilvD* or *leuCD* transcription. IlvD activity was assessed from the *ilvD*::*TN* (JMB3966; parent), *ilvD*::*TN* Δ*sufT* (JMB4376), and the genetically complemented *ilvD*::*TN* Δ*sufT sufT*^+^ (JMB4375) strains carrying p*ilvD* (Panel B). LeuCD activity was assessed from the *leuC*::*TN* (JMB4397; parent), *leuC*::*TN* Δ*sufT* (JMB4383), and the genetically complemented *leuC*::*TN* Δ*sufT sufT*^+^ (JMB4382) strains carrying p*leuCD* (Panel C). In p*ilvD* and p*leuCD* either *ilvD* or *leuCD* were under the transcriptional control of a xylose inducible promoter. The data represent the average of four (Panel A) or three (Panels B and C) biological replicates. Errors bars represent standard deviations and are shown in all panels but may not be visible where error is low.

Synthesis of the branched chain amino acids (BCAA) leucine and isoleucine requires the FeS cluster containing dehydratase enzymes isopropylmalate isomerase (LeuCD) and dihydroxyacid dehydratase (IlvD), respectively [60,61]. Strains lacking either SufT or Nfu displayed growth defects in defined medium lacking leucine (Leu) and isoleucine (Ile) (hereafter 18AA glucose medium) (Fig 4A), but displayed a growth profile similar to WT in 20AA glucose medium (S1B Fig).

We constructed *leuC*::*TN*, *leuC*::*TN* Δ*sufT*, *leuC*::*TN* Δ*sufT sufT*+, *ilvD*::*TN*, *ilvD*::*TN* Δ*sufT*, and the *ilvD*::*TN* Δ*sufT sufT*+ strains carrying plasmids with either *leuCD* or *ilvD* under the transcriptional control of a xylose inducible promoter (p*leuCD* and p*ilvD*). The activities of LeuCD and IlvD were decreased in strains lacking SufT and these defects were restored by genetic complementation (Fig 4B and 4C). We concluded that SufT is utilized in the maturation of multiple FeS cluster requiring enzymes.

### The role of SufT in FeS cluster assembly is increased during respiratory growth, but it is dispensable during fermentative growth

**Fig 5:**
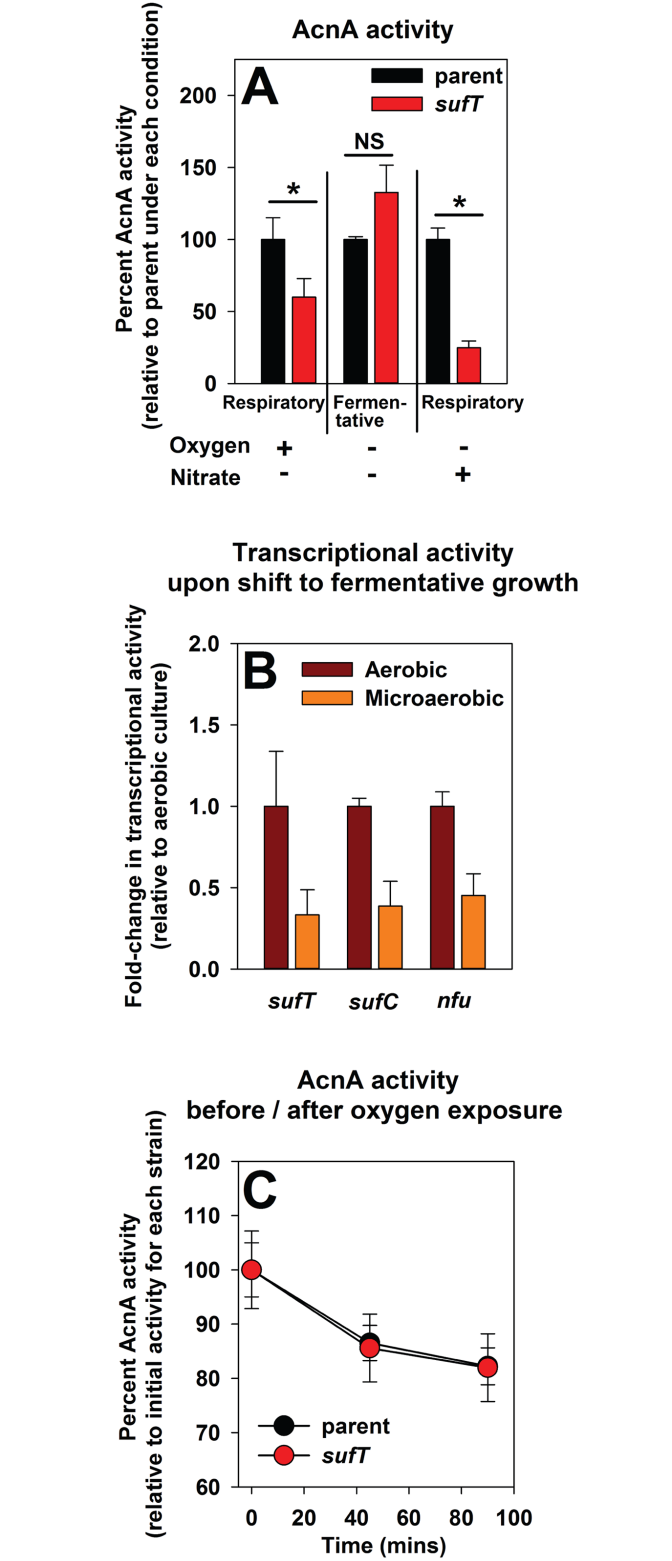
Delineating the role of SufT in FeS protein maturation during respiratory and fermentative growth. Panel A: A Δ*sufT* mutant has decreased AcnA activity during respiratory growth, but not during fermentative growth. AcnA activity was assessed from the *acnA*::*TN* (JMB3537; parent) and *acnA*::*TN* Δ*sufT* (JMB3539) strains containing p*acnA*. Strains were pre-cultured aerobically or anaerobically in the presence or absence of nitrate. Panel B: The transcriptional activities of *sufT*, *sufC*, and *nfu* are decreased upon a shift to fermentative growth. The transcriptional activities of *sufT*, *sufC*, and *nfu* were assessed in the WT (JMB1100). Cells were cultured aerobically to exponential phase before one set of cultures was incubated fermentatively (anaerobically) for one hour while the other set was incubated aerobically. Panel C: Dioxygen exposure decreases AcnA activity at a similar rate in the parent and Δ*sufT* strains. Cell-free lysates generated from the *acnA*::*TN* (JMB3537; parent) and the *acnA*::*TN* Δ*sufT* (JMB3539) strains containing p*acnA* were exposed to dioxygen and AcnA activity was recorded before and after exposure. The data presented represent the average of three (Panels A and B) or two (Panel C) biological replicates. Error bars signify standard deviations and are shown in all panels, but may not be visible where error is low. Where indicated, Student t-tests (two tailed) were performed on the data and * denotes p< 0.05. NS denotes that the data are not statistically significant. Strains containing p*acnA* have *acnA* under the transcriptional control of a xylose inducible promoter.

*Staphylococcus aureus* is a facultative anaerobe and can respire upon dioxygen or nitrate as terminal electron acceptors or grow fermentatively [62]. The *acnA*::*TN* and *acnA*::*TN* Δ*sufT* strains containing p*acnA* were cultured aerobically, as well as anaerobically in the presence or absence of nitrate before determining AcnA activity. The Δ*sufT* mutant had lower AcnA activity during respiratory growth, but AcnA activity was restored during fermentative growth (Fig 5A). Microaerobic conditions also mitigated the growth defect of both the Δ*nfu* and Δ*sufT* strains in 18AA glucose medium (S3 Fig).

Fermentative growth imposes a decreased demand for FeS clusters [63]. By inference, fermentative growth should result in decreased transcription of genes encoding for FeS assembly factors. Consistent with this prediction, the transcriptional activities of *sufT*, *nfu*, and *sufC* decreased when aerobically cultured cells were shifted to an anaerobic (fermentative) environment (Fig 5B).

We examined whether SufT functions to protect the AcnA FeS cluster via physical exclusion of dioxygen. Cell-free lysates were generated from the *acnA*::*TN* and *acnA*::*TN* Δ*sufT* strains containing p*acnA*. AcnA activity was assessed at periodic intervals before and after exposure of lysates to dioxygen. Dioxygen exposure resulted in decreased AcnA activity in both the parent and Δ*sufT* mutant (Fig 5C), but the rate of decrease was statistically indistinguishable between the strains.

### A strain lacking SufT is deficient in FeS cluster assembly upon the re-entry of fermenting staphylococcal cells into an aerobic environment

**Fig 6:**
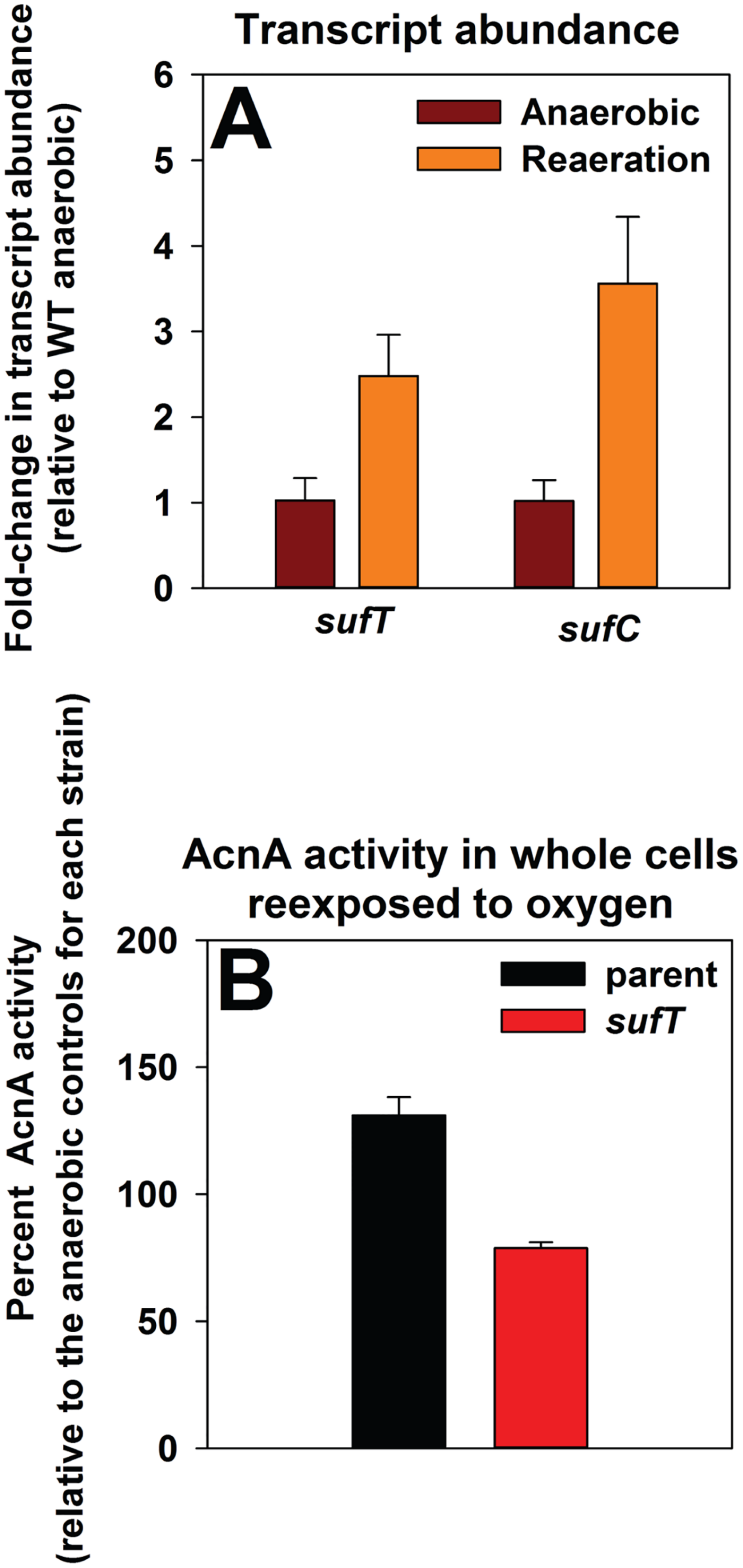
Examining the need for SufT upon exposure of fermenting cells to dioxygen. Panel A: Transcription of genes utilized for FeS assembly are increased when fermenting cells are exposed to dioxygen (reaeration). The WT strain (JMB1100) was cultured fermentatively for 4.5 hours before one set of cultures was exposed to dioxygen while the control cultures experienced continuous anaerobic growth. mRNA abundances corresponding to the *sufT* and *sufC* genes were assessed using quantitative RT-PCR. Data are presented as a ratio of transcript abundance upon dioxygen exposure to the abundance upon continued anaerobic incubation. The gene transcription profiles for the *sufC* gene upon reaeration were previously published [4]. Panel B: AcnA activity is decreased in the Δ*sufT* strain upon reaeration. The *acnA*::*TN* (JMB3537; parent) and the *acnA*::*TN* Δ*sufT* (JMB3539) strains containing p*acnA*, which contains *acnA* under the transcriptional control of a xylose inducible promoter, were cultured anaerobically before one set of cultures was exposed to dioxygen, while the control cultures experienced continuous anaerobic growth. The data in both panels represent the average of three biological replicates. Error bars signify standard deviations.

Fermentatively cultured cells exposed to dioxygen (reaeration) increased *sufC* transcription suggesting that the resumption of respiratory processes results in an increased demand for FeS clusters (Fig 6A and [4]). The transcription of *sufT* was also increased (~2.5-fold) upon reaeration (Fig 6A).

The role of SufT in the maturation of AcnA upon reaeration was assessed. The *acnA*::*TN* and *acnA*::*TN* Δ*sufT* strains containing p*acnA* were cultured fermentatively before one set of the cultures was exposed to dioxygen while the other set was incubated anaerobically (as previously described [40]). AcnA activity increased by ~30% in the parental strain upon dioxygen introduction (Fig 6B). In contrast, AcnA activity decreased by ~20% in the Δ*sufT* mutant. The use of protein synthesis inhibitors allowed for the conclusion that the increased AcnA activity in the parental strain upon reaeration was due to *de novo* protein synthesis. These findings led to the conclusion that SufT has a role in FeS cluster assembly in cells attempting to resume respiratory processes, and thereby facilitates the adaptation of cells to shifts in dioxygen tensions.

### A strain lacking SufT is deficient in FeS cluster assembly when cells experience ROS toxification, but is dispensable for physical protection from oxidants and the repair of H_2_O_2_ damaged FeS clusters

**Fig 7:**
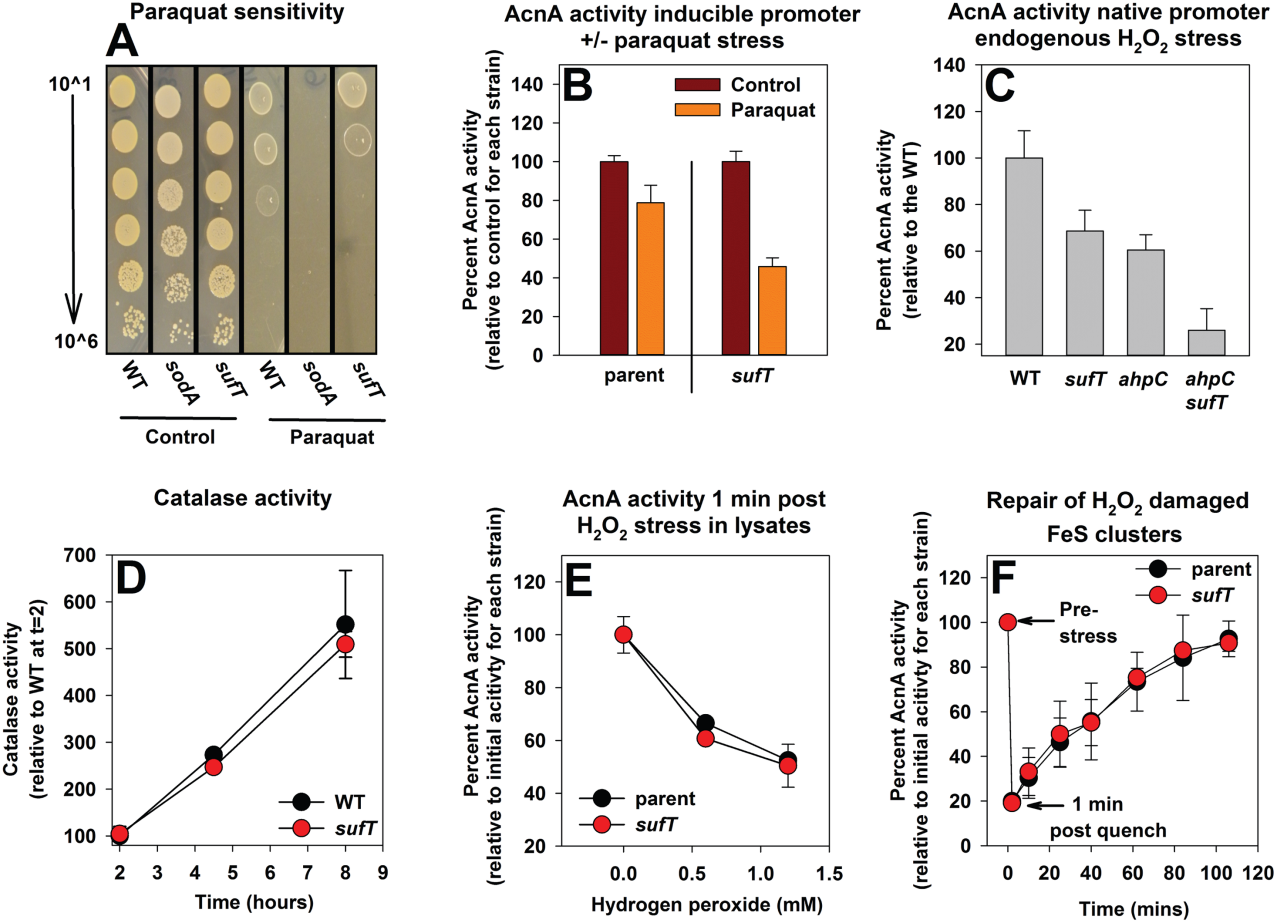
Delineating the role of SufT in FeS protein maturation upon ROS intoxication. Panel A: Strains lacking SufT or SodA are deficient for growth in the presence of paraquat. Growth of the WT (JMB1100), Δ*sufT* (JMB1146), and *sodA*::*TN* (JMB6326) strains cultured upon solid medium in the presence or absence of 30 mM paraquat. Panel B: A Δ*sufT* strain has decreased AcnA activity in cells challenged with paraquat. The *acnA*::*TN* (JMB3537; parent) and *acnA*::*TN* Δ*sufT* (JMB3539) strains containing p*acnA* were cultured aerobically to post exponential growth phase before one set of cultures was challenged with 40 mM paraquat for one hour and AcnA activity was determined. Panel C: A strain that is deficient in scavenging endogenous hydrogen peroxide and lacks SufT has decreased AcnA activity. AcnA enzyme activity was assessed from the WT (JMB1100), Δ*sufT* (JMB1146), *ahpC*::*TN* (JMB2080), and *ahpC*::*TN* Δ*sufT* (JMB6885) strains cultured aerobically. Panel D: Catalase activity is indistinguishable between the WT and Δ*sufT* strains across growth. Catalase activity was assessed from WT (JMB1100) and Δ*sufT* (JMB1146) strains. Kat activity was determined from the same cell-free lysates that were used to determine AcnA activity displayed in Fig 3B. Panel E: SufT is dispensable for the physical protection of AcnA from oxidant damage. Cell-free lysates were generated from the *acnA*::*TN* (JMB3537; parent) and the *acnA*::*TN* Δ*sufT* (JMB3539) strains containing p*acnA*. Lysates were treated with varying amounts of H_2_O_2_ and AcnA activity was determined one-minute post treatment. Panel F: SufT is dispensable for the repair of the [Fe_3_S_4_]^1+^ cluster of AcnA. Cell-free lysates were generated from the *acnA*::*TN* (JMB3537; parent) and the *acnA*::*TN* Δ*sufT* (JMB3539) strains containing p*acnA*. The lysates were exposed to 0.45 mM H_2_O_2_ for 1 minute before stress was terminated using catalase. AcnA activity was monitored before application of H_2_O_2_ and periodically after stress termination. The data represent the average of three (Panels B, C, and D) or two (Panels E and F) biological replicates. Error bars signify standard deviations. Strains containing p*acnA* have *acnA* under the transcriptional control of a xylose inducible promoter.

Reactive univalent species can damage or destroy solvent exposed FeS clusters [4,38,39]. We found that the Δ*sufT*, and *sodA*::*TN* (encoding for the dominant aerobic superoxide dismutase [64]) strains displayed decreased growth in the presence of paraquat, a redox cycling molecule that leads to increased accumulation of intracellular ROS (Fig 7A). However, the phenotype of the Δ*sufT* mutant was less severe than that of the *sodA*::*TN* strain.

The *acnA*::*TN* and *acnA*::*TN* Δ*sufT* strains containing p*acnA* were cultured, challenged with paraquat, and AcnA activity was determined. Challenging cells with paraquat resulted in ~15% and ~45% decrease in AcnA activity in the parent and Δ*sufT* mutant, respectively (Fig 7B).

The alkylhydroperoxidase system (Ahp) functions as an intracellular H_2_O_2_ scavenger and a *S*. *aureus* strain lacking Ahp accumulates intracellular ROS [4,65]. AcnA activity was assessed in the WT, Δ*sufT*, *ahp*::*TN*, and *ahp*::*TN* Δ*sufT* strains. AcnA activity was decreased ~25–30% in both the *ahp* and *sufT* strains and by ~75% in the *ahp sufT* double mutant strain (Fig 7C).

Four explanations could underlie the decreased AcnA activity observed in a Δ*sufT* strain upon ROS toxification: 1) the Δ*sufT* strain has decreased activities of ROS scavenging enzymes, 2) SufT is necessary for the repair of FeS clusters inactivated by ROS oxidation, 3) SufT is involved in physically shielding and/or excluding ROS from the enzyme active site and preventing damage, or 4) there is an increased need for SufT in FeS cluster assembly.

The activities of the ROS scavenging enzymes catalase (Kat) and superoxide dismutase (Sod) were similar in the WT and Δ*sufT* strains across growth (Fig 7D, S4 Fig). The *acnA*::*TN* and *acnA*::*TN* Δ*sufT* strains containing p*acnA* also displayed similar levels of Sod activity, both before and after paraquat treatment (S5 Fig).

We examined whether SufT is capable of physically shielding FeS clusters from univalent oxidants [43,44]. Cell-free lysates from the *acnA*::*TN* and *acnA*::*TN* Δ*sufT* strains containing p*acnA* were exposed to varying concentrations of H_2_O_2_ and AcnA activity was determined one minute post treatment. AcnA activity decreased with increasing H_2_O_2_ concentrations, but the decrease in AcnA activity was similar in the parent and Δ*sufT* mutant (Fig 7E).

Brief exposure to H_2_O_2_ can convert the active [Fe_4_S_4_]^2+^ cluster in AcnA into the inactive [Fe_3_S_4_]^1+^ cluster. This can be repaired to the [Fe_4_S_4_]^2+^ state by Fe^2+^ and an electron [40]. Cell-free lysates from the *acnA*::*TN* and *acnA*::*TN* Δ*sufT* strains containing p*acnA* were exposed to H_2_O_2_. One-minute post challenge, the stress was terminated and reactivation of AcnA activity by factors in the lysate was monitored over-time. The rate of AcnA reactivation was similar in the parent and Δ*sufT* mutant (Fig 7F). From Fig 7 we concluded that SufT is involved in the *de novo* assembly of FeS clusters in cells experiencing ROS stress.

### The role of SufT in FeS assembly increases in synchrony to the demand for FeS cluster containing proteins

The phenotypic abnormalities of the Δ*sufT* mutant were exacerbated during respiration, during resumption of respiration in fermenting cells, and upon ROS challenge (i.e. conditions imposing a high demand for FeS assembly). The transcription of core genes required for FeS assembly increased upon challenge with ROS or resumption of respiration [4].

**Fig 8:**
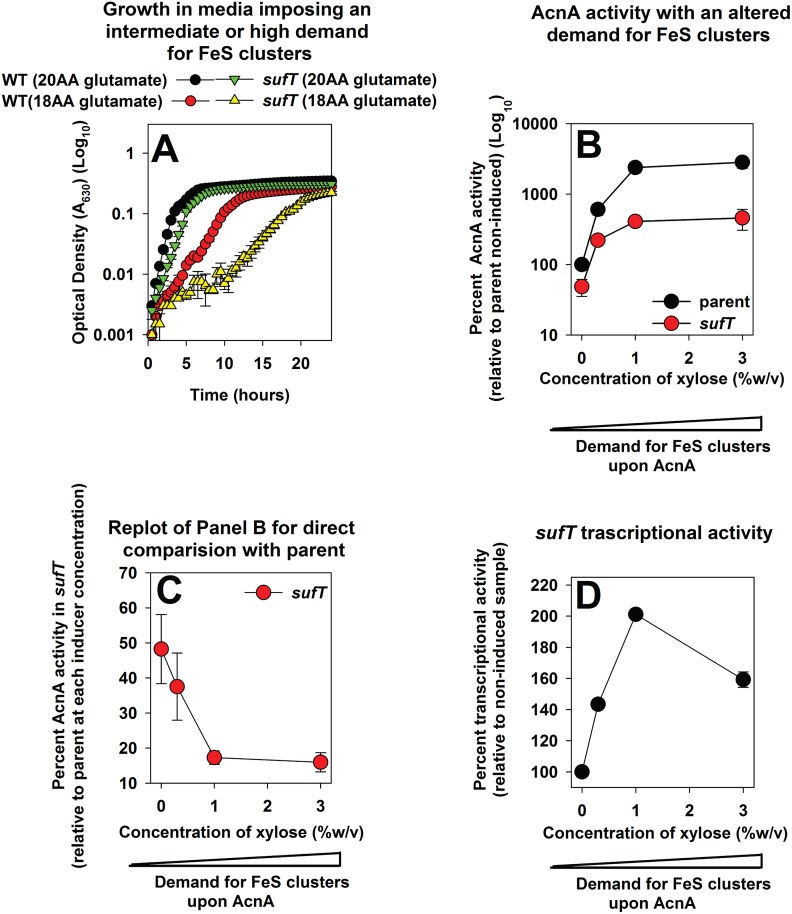
Examining the role of SufT in FeS protein maturation with respect to the demand for FeS clusters. Panel A: The demand for SufT for growth is increased in media that impose a high requirement for FeS proteins. Growth of the WT (JMB1100) and Δ*sufT* (JMB1146) strains in 20AA glutamate medium and 18AA glutamate medium. Panels B and C: The role of SufT in FeS assembly increases in synchrony with the demand for FeS clusters. The *acnA*::*TN* (JMB3537; parent) and the *acnA*::*TN* Δ*sufT* (JMB3539) strains containing p*acnA*, which contains *acnA* under the transcriptional control of a xylose inducible promoter, were cultured in media containing varying concentrations of xylose before AcnA activity was determined. Data were normalized with respect to the non-induced parent strain (Panel B) or with respect to the parent strain at each inducer concentration (Panel C). Panel D: Transcriptional activity of *sufT* increases in synchrony with the cellular demand for FeS clusters upon AcnA. The transcriptional activity for *sufT* was assessed in the *acnA*::*TN* (JMB3537) strain carrying p*acnA*, as well as a construct containing *gfp* under the transcriptional control of the *sufT* promoter. Cells were cultured in media containing varying concentrations of xylose. The data represent the average of three (Panels B and C) or two (Panels A and D) biological replicates. Error bars signify standard deviations.

We tested the hypothesis that SufT is required for FeS cluster assembly during conditions imposing a high demand for FeS clusters. Growth was monitored in either 20AA glutamate medium, or defined medium containing glutamate as a carbon source and lacking leucine (Leu) and isoleucine (Ile) (hereafter 18AA glutamate medium). Growth in 18AA glutamate medium would impose a simultaneous requirement for the AcnA, LeuCD, and IlvD enzymes, and by inference, exert an increased requirement for FeS clusters. The Δ*sufT* strain displayed a growth defect in 20AA glutamate medium (similar to Fig 3A; however the magnitude appears lower here due to the scale) and this defect was exacerbated upon culture in 18AA glutamate medium (Fig 8A).

The *acnA*::*TN* and *acnA*::*TN* Δ*sufT* strains containing p*acnA* were cultured in the presence or absence of varying concentrations of xylose followed by assessing AcnA activity. The difference in AcnA activity between the parent and Δ*sufT* mutant increased in synchrony with increasing inducer concentrations (Fig 8B and 8C).

We next monitored *sufT* transcriptional activity with respect to the demand for FeS clusters using the *acnA*::*TN* strain carrying p*acnA*, as well as the *sufT* transcriptional reporter. The transcriptional activity of *sufT* increased in synchrony with increasing inducer concentrations (Fig 8D).

### The DUF59 containing protein from *Mycobacterium tuberculosis* is able to rescue a growth defect of the *S*. *aureus* Δ*sufT* mutant

**Fig 9:**
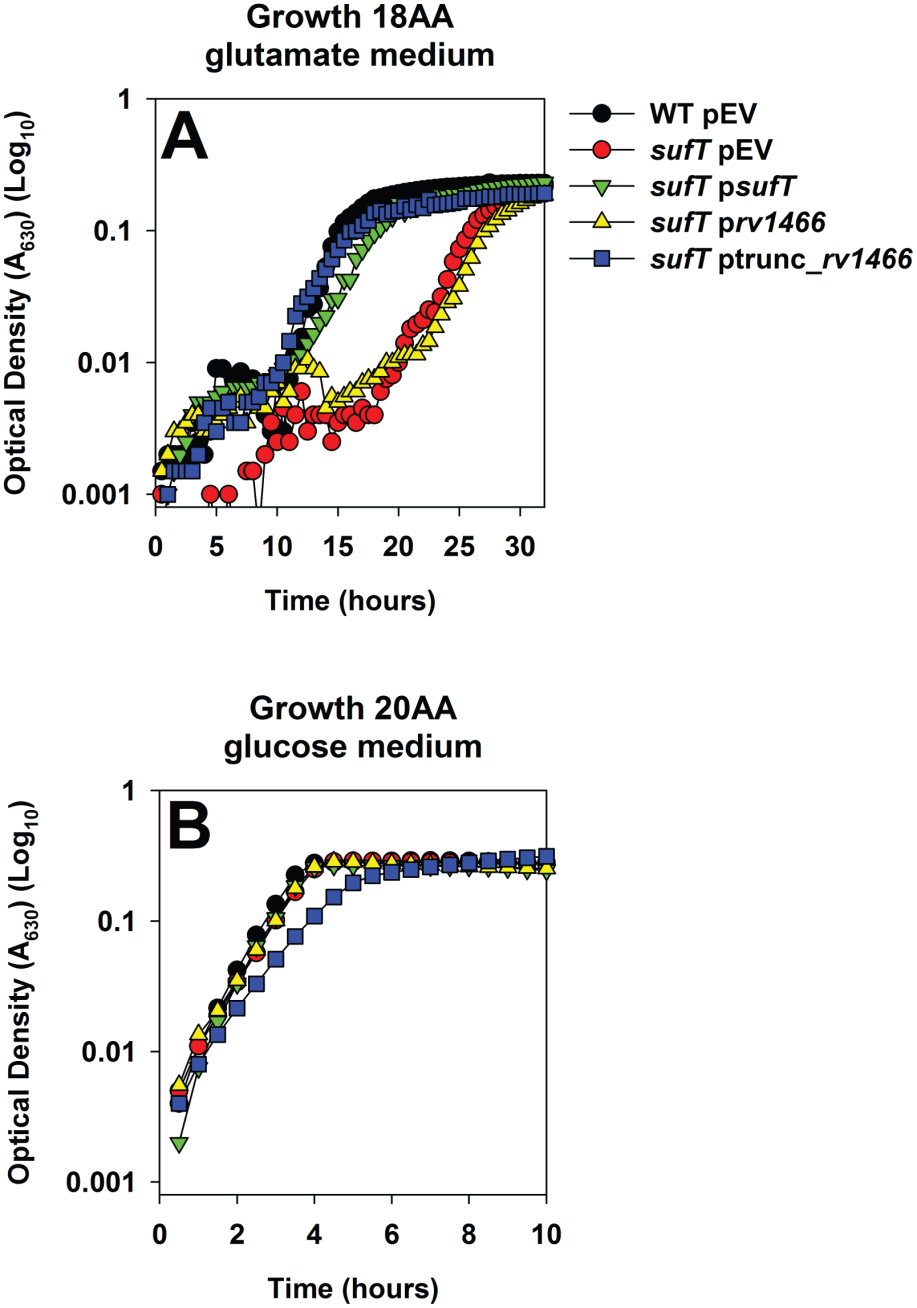
Functionality of the *Mycobacterium tuberculosis* DUF59 protein Rv1466 in *S*. *aureus*. Growth of the WT and Δ*sufT* strains carrying empty vector (pCM28) and the Δ*sufT* strain carrying pCM28_*rv1466* or pCM28_trunc_*rv1466* in 18AA glutamate medium (Panel A) or 20AA glucose medium (Panel B) are shown. The data represent the average of two biological replicates. Error bars signify standard deviations.

*Mycobacterium tuberculosis* contains a DUF59 containing protein (Rv1466) that is part of the *suf* operon and is essential for viability (Fig 1C and [66]). We examined whether Rv1466 could compensate for the loss of SufT in *S*. *aureus*. Rv1466 has a ~20 amino acid N-terminal extension when compared to the *S*. *aureus* SufT. Codon-optimized *rv1466* and a truncated version of *rv1466* (trunc_*rv1466*) were introduced upon a multi-copy plasmid into the *S*. *aureus* Δ*sufT* strain and phenotypes were examined. The presence of trunc_*rv1466*, but not *rv1466*, rescued the growth defect of the Δ*sufT* strain in 18AA glutamate medium (Fig 9A). The presence of trunc_*rv1466*, but not *rv1466*, displayed a dominant effect and inhibited growth of the Δ*sufT* strain in 20AA glucose medium (Fig 9B).

### *sufA* is epistatic to both *nfu* and *sufT*, while *nfu* and *sufT* display synergy

**Fig 10:**
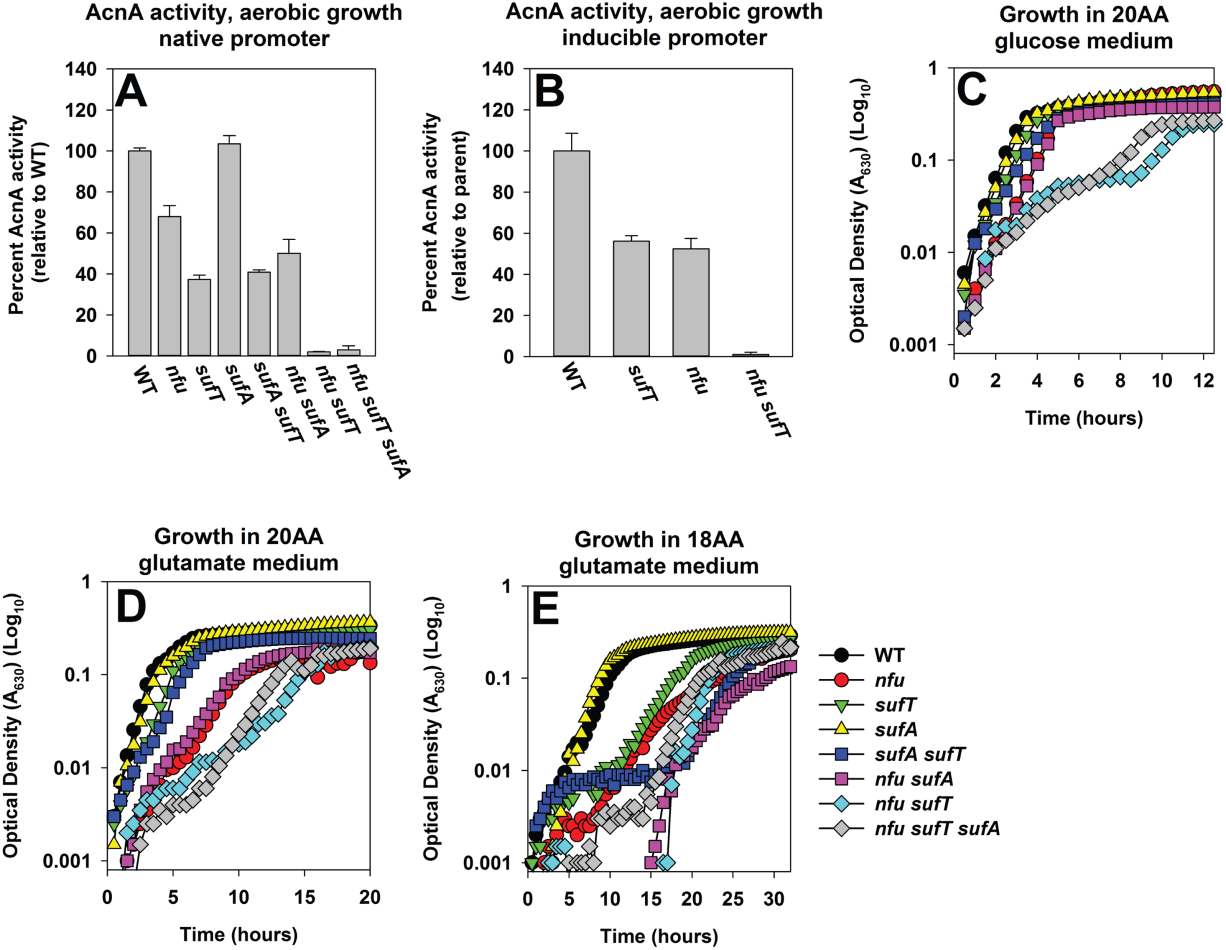
Epistatic relationships amongst auxiliary FeS cluster assembly factors. Panel A: *sufA* is epistatic to *nfu* while *nfu* and *sufT* display synergy with respect to AcnA activity. AcnA activity was assessed for the WT (JMB1100), Δ*sufT* (JMB1146), Δ*nfu* (JMB1165), Δ*nfu* Δ*sufT* (JMB2514), Δ*nfu* Δ*sufT* Δ*sufA* (JMB6835), Δ*sufA* (JMB2223), Δ*sufA* Δ*sufT* (JMB2224) and Δ*nfu* Δ*sufA* (JMB6834) strains. Panel B: The decreased AcnA activity of the *nfu sufT* strain is independent of *acnA* transcription. The *acnA*::*TN* (JMB3537; parent), *acnA*::*TN* Δ*sufT* (JMB3539), *acnA*::*TN* Δ*nfu* (JMB3538) and the *acnA*::*TN Δnfu* Δ*sufT* (JMB 7116) strains containing p*acnA*, which contains *acnA* under the transcriptional control of a xylose inducible promoter, were cultured aerobically and AcnA activity was determined. Panels C, D and E: *sufA* is epistatic to *nfu* and *sufT*, while *nfu* and *sufT* display synergy during growth. Growth of the strains used in panel A in 20AA glucose medium (Panel C), 20AA glutamate medium (Panel D), and 18AA glutamate medium (Panel E) are shown. The data represent the average of three (Panels A and B) or two (Panels C, D and E) biological replicates. Error bars signify standard deviations.

Epistatic relationships between *sufT*, *nfu*, and *sufA* were investigated by phenotypically examining mutant strains lacking one, two, or all three maturation factors. The Δ*sufA* strain did not display a defect in AcnA activity, relative to the WT strain, and the Δ*sufA* Δ*sufT* double mutant phenocopied the Δ*sufT* strain (Fig 10A). The phenotypic effects of the Δ*sufA* and Δ*nfu* mutations displayed an additive effect. AcnA activity in the Δ*nfu* mutant was ~65% of WT while the activity in the Δ*sufA* Δ*sufT* double mutant was ~50%. AcnA activity was near the limit of detection in the Δ*nfu* Δ*sufT* double mutant (~2%). The Δ*nfu* Δ*sufT* Δ*sufA* triple mutant had AcnA activity similar to the Δ*nfu* Δ*sufT* strain. AcnA activity in the *acnA*::*TN* Δ*nfu* Δ*sufT* strain containing p*acnA* was also nearly undetectable relative to its isogenic parental strains (Fig 10B). This suggested that the low AcnA activity in the Δ*nfu* Δ*sufT* strain was not solely the outcome of decreased *acnA* transcription.

Growth was examined in media that impose varying demands for FeS proteins (20AA glucose, 20AA glutamate, or 18AA glutamate media). The Δ*sufA* strain did not display a growth deficiency in any of the media examined (Fig 10C–10E). The Δ*sufA* Δ*sufT* double mutant phenocopied the Δ*sufT* strain in 20AA glucose and 20AA glutamate medium, but the effects of the mutations were additive in 18AA glutamate medium. The Δ*nfu* Δ*sufA* double mutant phenocopied the Δ*nfu* strain in 20AA glucose and 20AA glutamate media, but the effect of the mutations were additive in 18AA glutamate medium. The phenotypes of the Δ*nfu* and Δ*sufT* mutations displayed synergism. The Δ*nfu* Δ*sufT* double mutant displayed a severe growth defect in each media examined. The Δ*nfu* Δ*sufT* Δ*sufA* triple mutant strain largely phenocopied the Δ*nfu* Δ*sufT* strain in each media.

The Δ*nfu* Δ*sufT* double mutant also displayed severe growth defects in complex medium. Growth of *S*. *aureus* in tryptic soy broth (TSB) results in the consumption of glucose, the release of fermentative byproducts such as acetate, and acidification of the medium [67,68] followed by the uptake of the fermentative byproducts resulting in alkalization of the growth medium. Therefore, the pH and acetate profile of the spent medium correlates with the cells ability to uptake and utilize fermentation products [67,68,69]. We monitored optical densities, pH of the spent medium, and acetate concentrations in the spent medium over time in cultures of the WT, Δ*acnA*, Δ*nfu*, Δ*sufT*, and Δ*nfu* Δ*sufT* strains. The Δ*nfu* Δ*sufT* double mutant and Δ*acnA* strains displayed pronounced differences during post-exponential growth reaching lower final optical densities (S6A Fig). The pH of the medium from the Δ*nfu* Δ*sufT* and Δ*acnA* mutants did not re-alkalinize (S6B Fig) nor was acetate utilized (S6C Fig).

### *nfu* in multicopy partially mitigates the phenotypes of the Δ*sufT* mutant

The interactions amongst *sufT*, *nfu*, and *sufA* were further examined by introducing each gene upon a multi-copy plasmid (p*sufT*, p*nfu* and p*sufA*, respectively) and assessing whether they impart phenotypic suppression to the Δ*sufT* or Δ*nfu* strains. The Δ*sufA* strain did not have decreased AcnA activity, and therefore, suppression was not examined in this strain.

**Fig 11:**
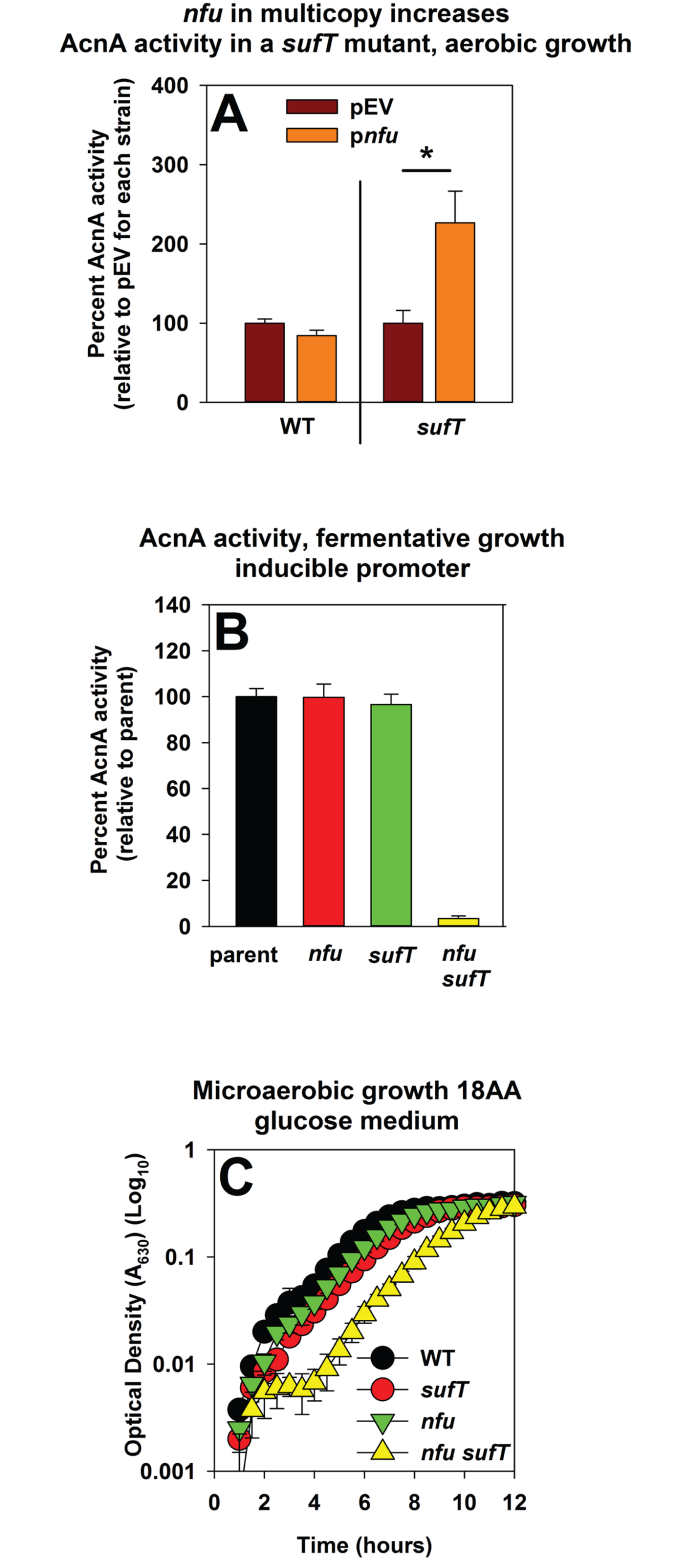
Examining genetic interactions between *nfu* and *sufT*. Panel A: AcnA activity is increased in a Δ*sufT* mutant strain carrying *nfu* upon a multicopy plasmid. AcnA activity was assessed from the WT (JMB1100) and Δ*sufT* (JMB1146) strains carrying either pEPSA5 (empty vector; pEV) or pEPSA5_*nfu* (p*nfu*) and cultured aerobically in medium containing 0.5% xylose to induce *nfu* transcription. Panel B: The phenotypes of the *nfu* and *sufT* mutations are synergistic with respect to AcnA activity during fermentative growth. The *acnA*::*TN* (JMB3537; parent), *acnA*::*TN* Δ*sufT* (JMB3539), *acnA*::*TN* Δ*nfu* (JMB3538) and the *acnA*::*TN Δnfu* Δ*sufT* strains containing p*acnA* were cultured fermentatively before AcnA activity was determined. Panel C: The phenotypes of the *nfu* and *sufT* mutations display synergy during microaerobic growth in 18AA glucose medium. Growth traces of the WT (JMB1100), Δ*sufT* (JMB1146), Δ*nfu* (JMB1165) and the Δ*nfu ΔsufT* (JMB2514) strains cultured microaerobically are shown. The data represent the average of four (Panel C) or three (Panels A and B) biological replicates. Error bars signify standard deviations. Where indicated, Student t-tests (two tailed) were performed on the data and * denotes p< 0.05.

The presence of p*sufA* appeared to increase AcnA activity mildly in both the WT and Δ*sufT* strains, but a statistically significant phenotypic rescue was not observed (S7A Fig). AcnA activity decreased in the Δ*nfu* strain carrying p*sufA*. AcnA activity was increased in the Δ*sufT* strain carrying p*nfu* (increase of ~250%), while the presence of p*nfu* had little effect on AcnA activity in the WT (Fig 11A). The presence of p*sufT* slightly decreased AcnA activity in the WT, while it did not alter AcnA activity in the Δ*nfu* strain (S7B Fig).

Growth profiles of the WT and Δ*sufT* strains carrying empty vector or p*nfu* were examined in 20AA glutamate medium. The presence of p*nfu* partially mitigated the growth defect of the Δ*sufT* strain in 20AA glutamate medium (S8 Fig).

### The presence of either Nfu or SufT is sufficient to maturate AcnA during conditions of low FeS cluster demand

The phenotypes of the Δ*sufT* strain were mitigated during fermentative growth, which imposes a low demand for FeS clusters. We reasoned that Nfu is utilized to fulfill the demand for FeS cluster assembly in the Δ*sufT* strain during fermentative growth. After fermentative culture the *acnA*::*TN* Δ*nfu* Δ*sufT* strain containing p*acnA* displayed levels of AcnA activity that were near the limit of detection (~2%), whereas the *acnA*::*TN* Δ*sufT* and *acnA*::*TN* Δ*nfu* strains had AcnA activity similar to the parent (Fig 11B). Microaerobic growth in 18 AA glucose medium was also examined. The Δ*nfu* and Δ*sufT* strains displayed growth profiles that did not significantly deviate from that of the WT (Fig 11C). However, the Δ*nfu* Δ*sufT* double mutant displayed a large growth defect.

From Figs 10 and 11, S7 and S8 Figs, we concluded that 1) the phenotypic effects of the *nfu* and *sufT* mutations are synergistic, 2) overproduction of *nfu* partially alleviates the phenotypes of the Δ*sufT* strain, and 3) either Nfu or SufT is sufficient for AcnA maturation during fermentative growth.

### Defective maturation of FeS proteins results in increased biofilm formation and decreased exoprotein production

**Fig 12:**
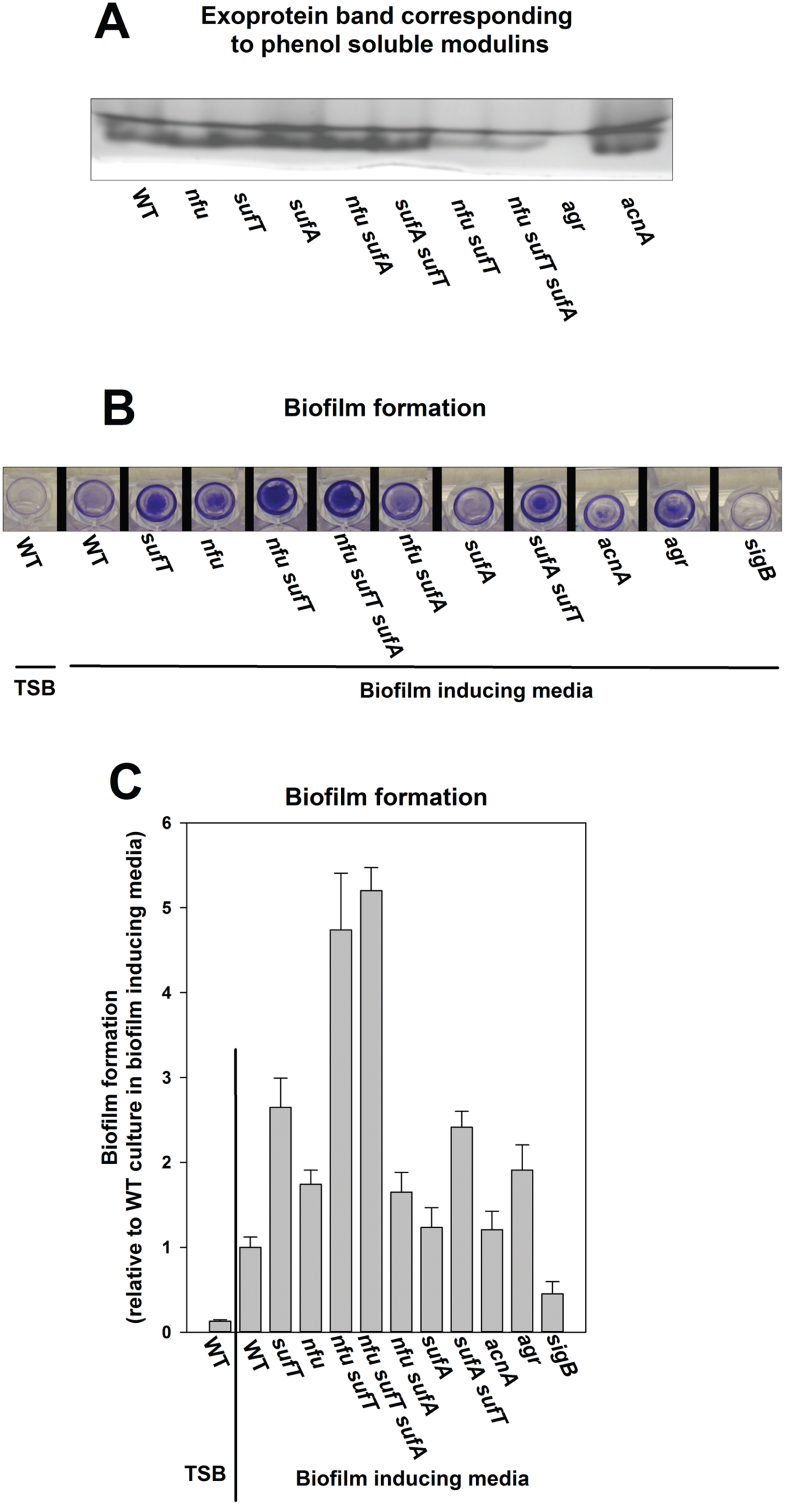
Biofilm formation and exoprotein production in strains defective in FeS cluster assembly. Exoprotein production (Panel A) and biofilm formation (Panel B and C) was assessed for the WT (JMB1100), Δ*sufT* (JMB1146), Δ*nfu* (JMB1165), Δ*nfu* Δ*sufT* (JMB2514), Δ*nfu* Δ*sufT* Δ*sufA* (JMB6835), Δ*sufA* (JMB2223), Δ*sufA* Δ*sufT* (JMB2224), Δ*nfu* Δ*sufA* (JMB6834) strains. For data in Panel A, spent medium supernatant from three biological replicates was standardized and combined, prior to precipitation and SDS-PAGE analyses. The data in panel C represent the average value of biofilms formed in eight independent wells. Error bars signify standard deviations. Representative photographs of biofilms formed on the surface of a 96-well microtiter plate are displayed in Panel B.

Biofilm formation and exoprotein production were assessed in strains lacking FeS cluster assembly factors. Agr is the dominant activator for transcription of exoproteins and toxins, as well as the phenol soluble modulins (PSMs). Therefore, an Δ*agr* strain was included as a positive control [51]. A strain lacking AcnA has been proposed to have increased Agr activity [70]. Since a Δ*nfu* Δ*sufT* strain phenocopied the *acnA*::*TN* mutant, the *acnA*::*TN* strain was also examined. Exoproteins were extracted from the spent medium supernatant and analyzed using SDS-PAGE. *S*. *aureus* encodes for eight PSMs that are small peptides comprising ~60% of the total exoproteome and are visualized on SDS-PAGE as one band [51]. The Δ*nfu* Δ*sufT*, Δ*nfu* Δ*sufT* Δ*sufA*, and the Δ*agr* strains were deficient in exoprotein production (Fig 12A). For ease of comparative analyses, only the band corresponding to PSMs is displayed.

Static growth of WT in TSB does not induce biofilm formation, and therefore, biofilm formation was examined in biofilm inducing medium (Fig 12B and 12C, [71]). Biofilm formation was also assessed in strains lacking Agr and SigB, which negatively and positively influence biofilm formation, respectively [72,73]. Strains deficient in the maturation of FeS proteins displayed varying degrees of biofilm formation. The Δ*nfu* Δ*sufT* double mutant displayed the largest increase in biofilm formation (~4.5 fold). The *acnA*::*TN* strain formed biofilms at a similar extent as the WT (Fig 12B and 12C).

### Defective maturation of FeS proteins imparts VISA-like phenotypes upon an otherwise vancomycin-susceptible MRSA isolate

**Fig 13:**
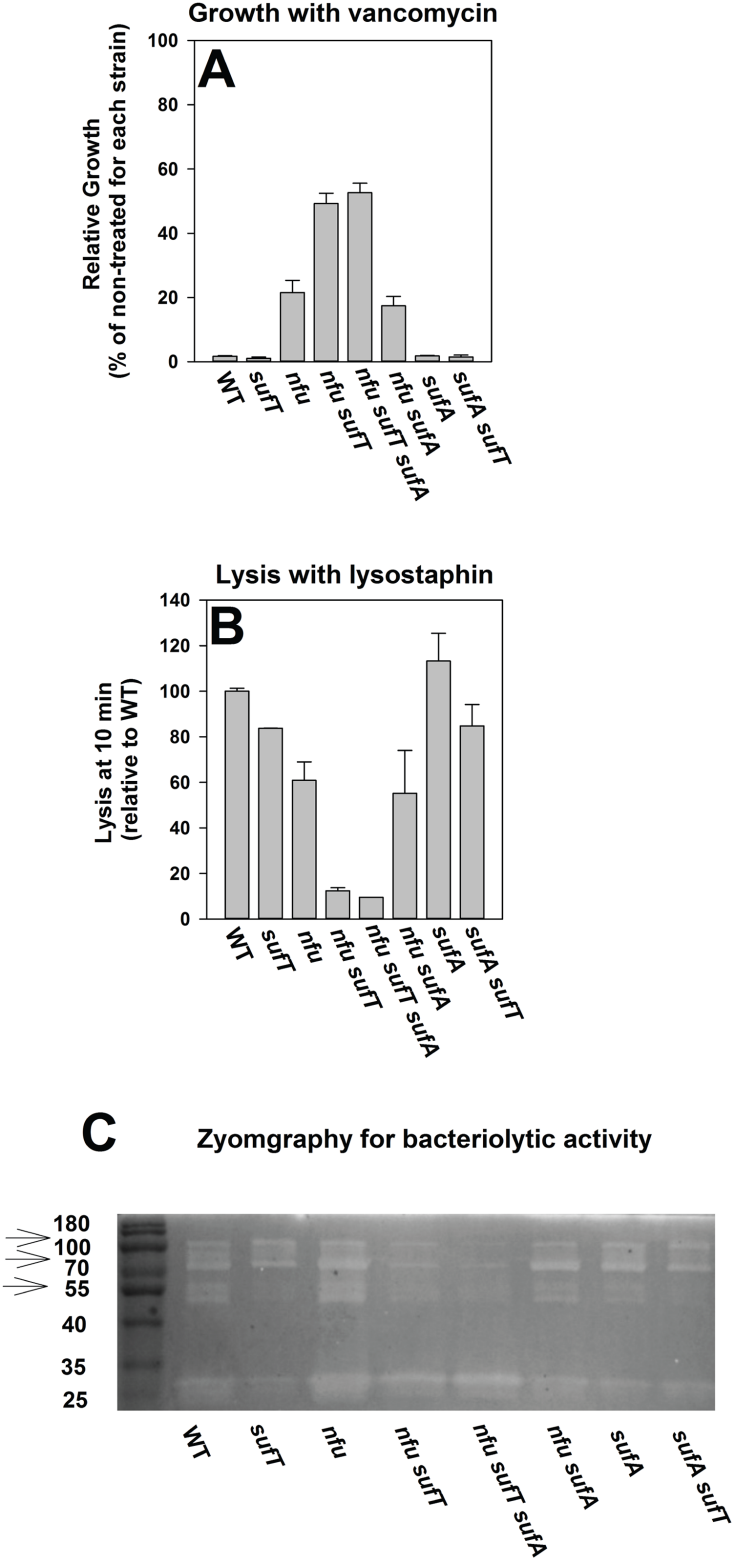
Examination of vancomycin resistance and VISA-like phenotypes in strains defective in FeS cluster assembly. Growth inhibition in the presence of vancomycin (Panel A), lysis of cells in the presence of lysostaphin (Panel B), and zymographic analyses of spent medium supernatant upon gels containing heat-killed WT cells as a substrate (Panel C) were assessed for the WT (JMB1100), Δ*sufT* (JMB1146), Δ*nfu* (JMB1165), Δ*nfu* Δ*sufT* (JMB2514), Δ*nfu* Δ*sufT* Δ*sufA* (JMB6835), Δ*sufA* (JMB2223), Δ*sufA ΔsufT* (JMB2224), Δ*nfu* Δ*sufA* (JMB6834) strains. The data in panels A and B represent the average value of two biological replicates. For data in Panel C spent medium supernatant from three biological replicates was standardized and combined prior to zymographic analyses. The arrows in Panel C point to bands that are reduced in intensity in strains lacking both Nfu and SufT.

We examined vancomycin sensitivities of strains lacking FeS cluster assembly factors. The Δ*nfu* Δ*sufT* double mutant displayed a large increase in resistance towards vancomycin during growth (Fig 13A). In growth inhibition curves we found that the Δ*nfu* Δ*sufT* strain was not completely resistant towards vancomycin, but rather, it displayed an inhibition response more characteristic of vancomycin-intermediate resistant *Staphylococcus aureus* (VISA) (S9 Fig and [74]).

Vancomycin resistant strains display alterations in their cell walls resulting in increased resistance towards lysis by lysostaphin [55,74]. The Δ*nfu* Δ*sufT* double mutant displayed the greatest resistance towards lysis by lysostaphin (Fig 13B).

Decreased activity of peptidoglycan hydrolases is a hallmark of VISA strains [55,74]. Peptidoglycan hydrolase activity was monitored using zymographic analysis upon heat-killed WT cells as a substrate. The Δ*nfu* Δ*sufT* double mutant displayed the largest alterations in the activities of peptidoglycan hydrolases (Fig 13C).

## Discussion

*Staphylococcus aureus* SufT is composed solely of a DUF59 domain. Alternate proteins containing DUF59 domains participate in FeS cluster assembly, but the function(s) of the DUF59 domain itself has not been described [46,47,48]. The goals of this study were to determine if SufT has a role in FeS cluster assembly, and if so, begin to dissect its *in vivo* functional role.

Phylogenetic analyses found that *sufT* was recruited to the same chromosomal location as *sufBC*, and once recruited, it was largely retained. These findings suggested that *sufT* was recruited to the operon to refine the functionality of Suf-mediated FeS cluster assembly. Amongst the genomes analyzed, only five organisms encoded for SufT, but not the FeS cluster scaffolding proteins SufB, IscU, or NifU. The five organisms identified were lactobacilli and within these genomes the SufT homolog was located within apparent operons that encode known FeS cluster requiring proteins. The informatics and phylogenetic findings strongly suggested a role for SufT in FeS cluster assembly.

The *S*. *aureus* Δ*sufT* strain displayed physiological abnormalities consistent with SufT having a role in the maturation of FeS proteins. Further, the phenotypes of the *S*. *aureus* Δ*sufT* strain closely resembled those of a strain lacking the FeS cluster carrier Nfu [4]. Aside from a role in *de novo* FeS cluster assembly, alternate possibilities for the observed deficiencies manifest in the Δ*sufT* strain were considered. The Δ*sufT* strain did not have altered H_2_O_2_ or superoxide scavenging activities. SufT was not required for the physical exclusion of H_2_O_2_ from the AcnA active site or the repair of the H_2_O_2_ damaged FeS cluster upon AcnA. These findings suggested that SufT likely functions in the *de novo* assembly of FeS clusters upon apo-proteins.

Genes encoding for proteins with functional overlap often display synergistic (superadditive) phenotypic effects when the gene products are absent or non-functional [75]. The phenotypes associated with *nfu* and *sufT* were synergistic. This was most evident during fermentative growth where there is a lower demand for FeS clusters. The phenotypes of the Δ*nfu* and Δ*sufT* strains were nearly indistinguishable from the WT strain, but the Δ*nfu* Δ*sufT* double mutant displayed a large growth defect and exhibited AcnA activity near the limit of detection. Introduction of *nfu* in multicopy to the Δ*sufT* strain led to partial mitigation of the phenotypes of this strain. Taken together, these findings led to the conclusion that both SufT and Nfu function as non-essential, accessory factors in the maturation of FeS proteins. Lending further support to this conclusion, subsequent to our informatics analyses, the genome of *Oligotropha carboxidovorans* was sequenced and found to encode for a protein consisting of a fusion of the N-terminus of Nfu and SufT (Locus tag: OCA5_c02770).

SufT, Nfu, and SufA are auxiliary FeS cluster maturation factors leading to the question of why *S*. *aureus* encodes for three such factors. The simplest explanations are that there is a degree of specificity for each auxiliary factor with respect to their target apo-proteins or that they have different functions. Vinella *et al*. have recently proposed an expanded model, which visualizes a dynamic cellular network of proteins that varies with growth stage or growth condition allowing for rapid calibration to alterations in the cellular demand for FeS protein maturation [76]. During such a scenario, certain auxiliary proteins and pathways would be preferred during normal growth and alternate auxiliary proteins and pathways during stress conditions.

The findings presented herein are consistent with the model proposed by Vinella *et al*. [76]. During routine aerobic growth, Nfu was the dominant auxiliary factor required for the maturation of AcnA. However, upon the overproduction of AcnA, the need for SufT for AcnA maturation was increased. The cellular need for SufT was also increased when cells were resuming respiration, toxified with ROS, or grown in 18AA glutamate medium; three conditions that impose a high demand for *de novo* FeS cluster assembly. The transcriptional activity of *sufT* also increased as the cellular demand for FeS clusters increased. These findings lend strong support to a model wherein SufT is a dominant factor involved in the maturation of FeS proteins in cells experiencing a high demand for FeS clusters. The epistasis experiments further strengthen the idea that certain accessory proteins are preferentially utilized when confronted with a high demand for FeS clusters. SufA was dispensable for growth under all conditions tested. However, SufA dependent phenotypes were manifest in strains lacking either Nfu or SufT and simultaneously cultured upon a medium imposing a high demand for FeS proteins. Therefore, we propose that SufA facilitates FeS protein maturation in *S*. *aureus* under conditions imposing a very high demand for FeS clusters. It is tempting to speculate that cells encode for multiple accessory maturation factors to respond to a gradation of demand for FeS cluster assembly, however, this awaits further experimentation.

It is currently unclear what genetic or biochemical elements dictate the increased usage of SufT or SufA upon increased FeS cofactor demand. Possible explanations include different functionalities, increased stability of a particular factor under stress conditions, or an increased rate of FeS cluster synthesis or FeS protein maturation under select cellular conditions. A similar scenario has been described to exist between the Suf and Isc FeS cluster biosynthetic machineries. In *Escherichia coli*, Suf is preferred under ROS stress and Fe limiting conditions, whereas Isc is the preferred FeS assembly system during conditions imposed by routine laboratory cultivation [77,78].

What is the role of SufT in FeS cluster assembly? The genetic findings presented make it tempting to speculate that SufT functions in the carriage of FeS clusters, but further biochemical analyses will be necessary to make this conclusion. It also worth noting that the SufT homologues analyzed in Fig 1 contain only one strictly conserved cysteine residue. With the exception of monothiol glutaredoxins, described FeS cluster carriers contain two or more cysteines utilized in FeS cluster ligation [79].

Biofilm formation and exoprotein production are critical in the infectious lifecycle of *S*. *aureus* [49,50]. We previously found that a strain lacking Nfu is attenuated for virulence in models of infection [4]. In this report we found that a strain that was crippled in its ability to maturate FeS proteins displayed significantly increased biofilm formation and decreased exoprotein production. Vancomycin is a last resort drug in the treatment of CA-MRSA infections and the genetic and molecular mechanisms underlying resistance to vancomycin are an active area of research [54]. Strains defective in FeS protein maturation also displayed an intermediate resistance to vancomycin and multiple phenotypes associated with VISA strains.

The Δ*nfu* Δ*sufT* strain phenocopied a Δ*acnA* strain in growth experiments, but it did not phenocopy this strain in phenotypes involved in virulence. *S*. *aureus* encodes for the FeS cluster utilizing two-component regulatory system (TCRS) AirSR [5]. AirSR alters the transcription of genes encoding for peptidoglycan hydrolases, as well as those required for biofilm formation [5,80]. AirR directly binds to the promoter region of Agr [80]. AirSR is also implicated in vancomycin resistance and a strain lacking AirSR displays VISA like phenotypes [80]. Therefore, the accumulation of apo-AirSR in the Δ*nfu* Δ*sufT* strain may underlie the virulence phenotypes witnessed. An alternate explanation is that the altered Agr activity in these strains results in altered virulence phenotypes. Apart from its roles in toxin production and biofilm formation, Agr has also been implicated in modulating vancomycin resistance in *S*. *aureus* [51,81,82]. Regardless of the mechanism(s) underlying the phenotypes presented, these findings highlight the importance of efficient FeS cluster assembly for multiple phenotypes critical for pathogenesis and antibiotic resistance.

In summary, we have identified a role for SufT, and by extension DUF59, in the maturation of FeS proteins. We propose a model wherein SufT is an auxiliary FeS protein maturation factor whose usage is selectively increased during growth conditions necessitating increased FeS cluster assembly in *S*. *aureus*. An increased demand for FeS clusters may have been an evolutionary driving force to recruit *sufT* to the *suf* operon thereby increasing the efficiency and control of *de novo* FeS cluster assembly.

## Materials and Methods

### Materials

Restriction enzymes, quick DNA ligase kit, deoxynucleoside triphosphates, and Phusion DNA polymerase were purchased from New England Biolabs (Ipswich, MA). The plasmid mini-prep kit, gel extraction kit and RNA protect were purchased from Qiagen (Hilden, Germany). Lysostaphin was purchased from Ambi products (Lawrence, NY). Oligonucleotides were purchased from Integrated DNA Technologies (Coralville, IA) and sequences are listed in S1 Table (oligonucleotides used in this study). Trizol (Life Technologies), High-Capacity cDNA Reverse Transcription Kits (Life Technologies), and DNase I (Ambion) was purchased from Thermo Fisher Scientific (Waltham, MA). Tryptic Soy Broth (TSB) was purchased from MP Biomedicals (Santa Ana, CA). An acetic acid quantification kit was purchased from R-BioPharma (Darmstadt, Germany). Unless specified all chemicals were purchased from Sigma-Aldrich (St. Louis, MO) and were of the highest purity available.

### Bacterial growth conditions

Unless otherwise stated, the *S*. *aureus* strains used in this study (listed in Table 1) were constructed in the *S*. *aureus* community-associated USA300 strain LAC that was cured of the native plasmid pUSA03, which confers erythromycin resistance [83]. The USA300 LAC genome differs from USA300_FPR3757 only by a few single nucleotide polymorphisms [84,85]. Unless specifically mentioned, *S*. *aureus* cells were cultured as follows: 1) aerobic growth at a flask/tube headspace to culture medium volume ratio (hereafter HV ratio) of 10; 2) anaerobic growth at a flask/tube headspace to culture medium volume ratio of 0, as described earlier [4]; 3) in 96-well microtiter plates containing 200 μL total volume (detailed procedure below). Liquid cultures were grown at 37°C with shaking at 200 rpm unless otherwise indicated. Difco BioTek agar was added (15 g L^-1^) for solid medium. When selecting for plasmids, antibiotics were added at the final following concentrations: 150 μg mL^-1^ ampicillin (Amp); 30 μg mL^-1^ chloramphenicol (Cm); 10 μg mL^-1^ erythromycin (Erm); 3 μg mL^-1^ tetracycline (Tet); 125 μg mL^-1^ kanamycin (Kan); 150 ng mL^-1^ anhydrotetracycline (Atet). For routine plasmid maintenance, liquid media were supplemented with 10 μg mL^-1^ or 3.3 μg mL^-1^ of chloramphenicol or erythromycin, respectively.

**Table 1:** Strains and plasmids used in this study^a^.

Strains used in this study			
*S*. *aureus* Strains	Genotype/Description	Genetic Background	Source/ Reference
JMB1100	USA300_LAC (erm sensitive)	LAC	[83]
RN4220	Restriction minus	NCTC8325	[104]
JMB2316	*Δnfu*::*tetM*	LAC	[4]
JMB1165	*Δ*SAUSA300_0875(*nfu)*	LAC	[4]
JMB1146	*Δ*SAUSA300_0875(*sufT*)	LAC	This work
JMB6326	*sodA*::*TN*(*ermB*) (SAUSA300_1513)	LAC	NARSA [105]
JMB2080	*ahpC*::*TN*(*ermB*) (SAUSA300_0379)	LAC	V. Torres
JMB6885	*ΔsufT ahpC*::*TN*(*ermB*)	LAC	This work
JMB1144	*Δ*SAUSA300_0843(*sufA*)	LAC	[4]
JMB2224	*ΔsufA*::*tetM ΔsufT*	LAC	This work
JMB2514	*ΔsufT nfu*::*tetM*	LAC	This work
JMB1580	*Δnfu*::*kanR*	LAC	[4]
JMB2223	*ΔsufA*::*tetM*	LAC	[4]
JMB6834	*ΔsufA*::*tetM Δnfu*::*kanR*	LAC	This work
JMB6835	*ΔsufA*::*tetM Δnfu*::*kanR ΔsufT*	LAC	This work
JMB1977	*Δagr*::*tet*	LAC	[72]
JMB1102	*ΔsigB*	LAC	[97]
NE892	SAUSA300_2012(*leuC*)::*TN*(*ermB*)	LAC	NARSA [105]
NE718	SAUSA300_2006(*ilvD*)::*TN*(*ermB*)	LAC	NARSA [105]
JMB1432	*Δfur*::*tet*	LAC	[106]
JMB1163	*ΔacnA*::*tet*	LAC	[107]
JMB3537	*acnA*::*TN*(*ermB*)	LAC	[4]
JMB3538	*Δnfu acnA*::*TN*(*ermB*)	LAC	[4]
JMB3539	*ΔsufT acnA*::*TN*(*ermB*)	LAC	This work
JMB7116	*Δnfu* *ΔsufT*::*tetM acnA*::*TN*(*ermB*)	LAC	This work
JMB4432	*acnA*::*TN*(*ermB*), *attP*::*pLL39*	LAC	[4]
JMB4374	*ΔsufT*,*acnA*::*TN*(*ermB*), *attP*::*pLL39*	LAC	This work
JMB4373	*ΔsufT*, *acnA*::*TN*(*ermB*), *attP*::*pLL39_sufT*	LAC	This work
JMB4397	*attP*::*pLL39*, *leuC*::*TN*(*ermB*)	LAC	[4]
JMB4383	*ΔsufT*,*leuC*::*TN*(*ermB*), *attP*::*pLL39*	LAC	This work
JMB4382	*ΔsufT*,*leuC*::*TN*(*ermB*), *attP*::*pLL39_sufT*	LAC	This work
JMB3966	*ilvD*::*TN*(*ermB*), *attP*::*pLL39*	LAC	[4]
JMB4376	*ΔsufT*, *ilvD*::*TN*(*ermB*) *attP*::*pLL39*	LAC	This work
JMB4375	*ΔsufT*,*ilvD*::*TN*(*ermB*) *attP*::*pLL39_sufT*	LAC	This work
Other Strains			
*Escherichia coli* PX5			Protein Express
*Escherichia coli* BL21-AI*			Life Technologies
Plasmids used in this study			
Plasmid name	Insert locus/function		Source/Reference
pJB38	construction of chromosomal gene deletions		[108]
pJB38_*sufT*	Construction of Δ*sufT*		This work
pJB38_*sufT*::*tet*	Construction of *sufT*::*tet* allele		This work
pCM28	Cloning vector for genetic complementation		A. Horswill
pCM11	Cloning vector for transcriptional reporters		[109]
pCM11_*sufC*	Reporter construct transcriptional activity		This work
pCM11_*sufT*	Reporter construct transcriptional activity		This work
pCM11_*nfu*	Reporter construct transcriptional activity		This work
pCM11_*acnA*	Reporter construct transcriptional activity		This work
pCM28_*sufT*	Genetic complementation		This work
pCM28_*rv1466*	Genetic complementation		
pCM28_trunk_*rv1466*	Genetic complementation		
pLL39	Chromosomal genetic complementation		[110]
pLL39_*sufT*	Chromosomal genetic complementation		This work
pEPSA5	Multicopy genetic complementation		[111]
pLL2787	ϕ11 *int*		[110]
pDG783	*kanR*		[112]

^a^ Abbreviations: TN; transposon insertion.

### Strain and plasmid construction

*Escherichia coli* DH5α was used as a cloning host for plasmid constructions. All clones were passaged through RN4220 and transductions were conducted using phage 80α [86]. All *S*. *aureus* mutant strains and plasmids were verified using PCR or by sequencing PCR products or plasmids. All DNA sequencing was performed by Genewiz (South Plainfield, NJ).

Unless otherwise stated, JMB1100 chromosomal DNA was used as a template for PCR reactions. To create the Δ*sufT* deletion strain (JMB1146), approximately 500 base pairs upstream and downstream of *sufT* gene (SAUSA300_0875) were amplified using PCR with primer pairs 0875up5EcoRI and 0875up3NheI; 0875dwn5MluI and 0875 dwn3BamHI (S1 Table). Amplicons were gel purified and fused using PCR and the 0875up5EcoRI and 0875 dwn3BamHI primers. The resulting amplicon was gel purified, and digested with BamHI and SalI, followed by a ligation into similarly digested pJB38 resulting in pJB38_Δ*sufT*. The plasmid pJB38*_*Δ*sufT* was isolated and subsequently transformed into RN4220 before transducing into JMB1100. A single colony was inoculated into 5 mL of TSB-Cm and cultured overnight at 42°C followed by plating 25 μL on TSA-Cm to select for colonies containing a single recombination event. Single colonies were inoculated into 5 mL of TSB medium and were grown overnight, followed by a dilution of 1:25,000 before plating 100 μL onto TSA containing Atet to select against plasmid containing cells. Colonies were screened for Cm sensitivity and for the Δ*sufT* mutation using PCR.

The *sufT*::*tetM* strain was created by digesting the pJB38_ *sufTΔ* with MluI and NheI and inserting the *tetM* gene between the upstream and downstream regions of *sufT*. The DNA encoding for Tet resistance (*tetM*) was amplified using PCR with Strain JMB1432 as a template and the G+tetnheI and G+tetmluI primers before digesting and ligating into similarly digested pJB38_Δ*sufT*. The resulting plasmid (pJB38_Δ*sufT*::*tetM*) was passaged though *E*. *coli*, before it was transformed into RN4220. The Δ*sufT*::*tetM* mutant was constructed as described above.

Plasmids for genetic complementation, transcriptional analyses, and insertion of epitope tags to allow protein detection by western blots were constructed by subcloning digested PCR products into similarly digested vectors or by using yeast homologous recombination cloning (YRC) as previously described [87,88]. The pLL39_*sufT* and pCM28_*sufT* plasmids were created using the 0875_5BamHI and the 0875_3SalI primer pair. The pCM11_*sufT* was created using the 875gfpKpnI and 875gfpHindIII primer pair. The pCM11_*acnA* was made using the AcnApHindIII and AcnApKpnI primer pair. The *Mycobacterium tuberculosis rv1466* was codon optimized and synthesized by Integrated DNA technologies (IDT; Coralville, IA) and cloned into pCM28 using the native *S*. *aureus sufT* promoter using YRC. The full-length construct was constructed using amplicons generated using the following primer pairs: pCM28YCC and Ycc875p3; ycc875p5 and 875pMT3; 875pMT5 and 875pCM28 3. The truncated version was created using the same primers except MT875trunk5 and MT875trunk3 replaced ycc875p5 and Ycc875p3, respectively.

### Growth analyses

Growth was assessed in 200 μL cultures grown at 37°C in 96-well plates using a BioTek 808E Visible absorption spectrophotometer. Culture optical density was monitored at 630 nm. The staphylococcal-defined medium has been described previously [4]. Strains cultured overnight in TSB were inoculated into minimal medium or TSB to a final optical density (OD) of 0.025 (A_600_) units. For assessing nutritional requirements, cultures were harvested and treated as above, except that the cell pellet was washed twice to prevent carryover of rich medium components. For aerobic growth the shake speed was set to medium. For microaerobic growth the plate was incubated statically.

The four growth medium formulations utilized for nutritional analyses were: 1) 20AA glucose medium, containing the 20 canonical amino acids and 14 mM glucose as a source of carbon; 2) 18AA glucose medium, containing 18 canonical amino acids and lacking leucine and isoleucine and 14 mM glucose as a source of carbon; 3) 20AA glutamate medium, containing the 20 canonical amino acids and 44 mM glutamate as a source of carbon, and 4) 18AA glutamate medium, containing 18 canonical amino acids and lacking leucine and isoleucine and 44 mM glutamate as a source of carbon.

To examine vancomycin sensitivity, cultures were inoculated into TSB in the presence or absence of varying concentrations of vancomyin (0.025–1.5 μg/mL). Growth inhibition was assessed after 4 hours of growth. Paraquat sensitivity assays were conducted upon solid tryptic soy broth agar (TSA) plates containing 0 or 30 mM of paraquat. Overnight cultures (~18 hours of growth) were serial diluted in 1X phosphate buffered saline and 10 μL of each dilution was placed on plates of the solid medium. The plates were incubated at 37°C for 15 hours before the growth was assessed.

### Transcriptional reporter fusion assay

Strains cultured overnight in TSB-Erm medium were diluted into fresh TSB-Erm medium to a final OD of 0.1 (A_600_) and cultured, with shaking, at a HV ratio of 10. At periodic intervals culture density and fluorescence were assessed as described previously [4]. Fluorescence data were normalized with respect to a strain not carrying a GFP-based transcriptional reporter to normalize for background fluorescence values. The resulting data were normalized to the culture OD. Finally for ease of comparative analyses the data were normalized relative to the wild-type (WT) strain, or as specified in the figure legend.

Anaerobic culture conditions were achieved as described earlier [4,89]. Cells were cultured to exponential growth, aerobically, as described above. The cultures were then split and one set of cells was cultured at a HV ratio of zero in capped microcentrifuge tubes and anaerobiosis was verified by the addition of 0.001% resazurin to control tubes [4,89].

### RNA extractions and real time quantitative PCR (RT-PCR)

mRNA abundances of genes were examined from a previously described cDNA library [4].

### Cell-free extract enzyme assays

#### Aconitase (AcnA) assays

Strains cultured overnight in TSB were diluted into fresh TSB to a final OD of 0.1 (A_600_). For strains carrying p*acnA* the medium was amended with 1% xylose to induce gene transcription, unless specifically mentioned otherwise and cultured for 8 hours (~OD of 8) and at a HV ratio of either 10 or 0. The HV ratios were altered as per experimental requirements and details are mentioned in each figure legend. For strains where chromosomal levels of AcnA were assessed, strains were cultured for 18 hours at a HV ratio of 15.

For AcnA assays using anaerobically cultured *S*. *aureus*, strains were cultured in 2 mL microcentrifuge tubes containing 2 mL of culture medium (formulation as described above) and at a HV ratio of zero, as described earlier [4]. Anaerobic conditions were verified by the addition of 0.001% resazurin to control tubes and the medium color was monitored over time. Anaerobiosis was achieved by 3 hours post inoculation.

For assessing the effects of paraquat, cells were cultured to post exponential growth phase and one set of cultures was challenged with 40 mM paraquat for 1 hour, prior to harvest.

For assessing the effects of the reaeration (re-exposure of cells cultured anaerobically (fermentative growth) to dioxygen) in whole cells, strains were cultured anaerobically as described above for 4.5 hours. To induce reaeration, tubes were uncapped and rapidly transferred into shake tubes at a HV ratio of 15. Cultures were subsequently grown for 35 minutes with vigorous shaking prior to harvest.

Anaerobic growth of *S*. *aureus* cells upon sodium nitrate as a terminal electron acceptor results in the respiratory reduction of nitrate to nitrite. NO is an acidified nitrite derivative and can arise during respiration upon nitrate in cells cultured in TSB medium [90]. NO can inactivate FeS clusters [91,92]. Thus, in experiments where the effect of sodium nitrate is assessed, the growth medium (TSB) was buffered with 50 mM Hepes, pH 7.2 to prevent NO species formation.

To assess AcnA activity, cell pellets were harvested by centrifugation, placed inside a COY anaerobic chamber, and re-suspended in 100 μL anaerobic lysis buffer (50 mM Tris, 150 mM NaCl, pH 7.4). Cells were lysed by the addition of 4 μg lysostaphin and 8 μg DNAse and incubated at 37°C until confluent lysis was observed. The cellular lysates were clarified using a 10 minute high-speed spin. Lysates were removed from the anaerobic chamber and between 15 and 25 μL of lysate was added to 985–975 μL (total volume of 1 mL) of lysis buffer containing 20 mM DL-isocitrate. Aconitase activity was determined by monitoring the conversion of isocitrate to cis-aconitate spectrophotometrically using a Beckman Coulter DU530 UV-Vis absorption spectrophotometer (cis-aconitate ε240 nm = 3.6 mM^-1^cm^-1^ [93]). Enzymatic activity was standardized with respect to the total protein concentration and subsequently to that of the parental strain or as indicated in the figure legend.

The reactivation (repair) of hydrogen peroxide (H_2_O_2_) damaged FeS clusters upon AcnA was performed as described earlier, with minor modifications [41]. Cell-free lysates were generated from strains cultured anaerobically. At time zero the lysates were treated with 0.45 mM H_2_O_2_. After one minute, H_2_O_2_ stress was terminated by the addition of 45 μg/mL of catalase. FeS cluster repair was monitored by following recovery of AcnA activity over time.

To assess the effect of dioxygen upon AcnA *in vitro*, cell-free lysates were generated anoxically and time zero AcnA activity was recorded. Subsequently the lysates were exposed to dioxygen by incubation in 1.5 mL microcentrifuge vessels with the caps left open and shaking upon a thermomixer at 600 rpm and 37°C. AcnA activity were recorded periodically post dioxygen exposure.

#### Isopropylmalate isomerase (LeuCD) assays

Cells were cultured, harvested, and cell pellets obtained as described previously [4]. LeuCD activity was assayed following addition of 20 μL of lysate to 680 μL of buffer (50mM Tris, pH 8.0) containing 10 mM MgCl_2_ and 10 mM DL-Threo-3-isopropylmalic acid. LeuCD was assayed as a functional ability to convert 3-isopropylmalate to dimethylcitraconate acid spectrophotometrically (dimethylcitrateconate ε235nm = 4.35mM^-1^ cm^-1^), as described previously [94]. Enzymatic activity was standardized with respect to the total protein concentration and subsequently to that of the parental strain or as indicated in the figure legend.

#### Dihydroxy-acid dehydratase (IlvD) assays

Cells were cultured, harvested and cell pellets obtained as described previously [4]. IlvD activity was determined by the addition of 20 μL of cell-free extract to a buffer containing 50 mM Tris (pH 8.0) supplemented with 10 mM MgCl_2_ and 10 mM D,L-2,3-dihydroxy-isovalerate. Keto acid formation from D,L-2,3-dihydroxy-isovalerate was monitored spectrophotometrically (keto acids ε240nm = 0.19 mM^-1^ cm^-1^) to determine the activity of IlvD. Enzymatic activity was standardized with respect to the total protein concentration and subsequently to the activity of the parental strain or as indicated in the figure legend.

#### Catalase assays

Cells were cultured, harvested, and cell pellets obtained as described above for aconitase assays or as described in the figure legend. The cell lysate was further diluted 50-fold in lysis buffer and catalase activity was assayed by the addition of 5 μL of the diluted extract to 975 μL of assay buffer A (50 mM Tris, pH 7.5, 150 mM NaCl, and 18 mM H_2_O_2_). The decomposition of H_2_O_2_ was monitored spectrophotometrically, as described elsewhere [95].

#### Superoxide dismutase assays

Cells were cultured, harvested, and cell pellets obtained as described above for aconitase assays or as specified in the figure legend. SOD activity in the cell lysates was determined using the xanthine oxidase-cytochrome c method [96].

### Lysis with lysostaphin

Strains were cultured overnight in TSB and cells were harvested by centrifugation. Cell pellets were washed twice with 1X phosphate buffered saline and resuspended in lysis buffer (recipe above) in the presence of 5 μg/mL of lysostaphin. The lysostaphin mediated decrease in optical densities (A_600_) was recorded periodically.

### Protein concentration determination and western blot analyses

Protein concentration was determined using a copper/bicinchonic acid based colorimetric assay modified for a 96-well plate (47). Bovine serum albumin (2 mg/mL) was used as a standard. Western blot analyses were conducted as described previously [4,88].

### Determination of optical density, pH profiles and acetic acid concentration in spent medium

Strains cultured overnight in TSB (~18 hours) were diluted into fresh TSB to a final OD of 0.1 (A_600_). Periodically, aliquots of the cultures were removed, optical density was determined, and the cells and culture media were partitioned by centrifugation at 14,000 rpm for 1 minute. Two mL of either the culture supernatant or sterile TSB, which served to provide a pH reading for the point of inoculation, were combined with 8 mL of distilled and deionized water and the pH was determined using a Fisher Scientific Accumet AB15 pH mV Meter. The concentration of acetic acid in spent media was determined using the R-Biopharm Enzymatic BioAnalysis kit following the manufacturer's suggested protocol.

### Static model of biofilm formation

Biofilm formation was examined as described elsewhere, with minor changes [71,97]. Briefly, overnight cultures were diluted into biofilm media (TSB supplemented with 3% NaCl and 0.5% glucose), added to the wells of a 96-well microtiter plate and incubated statically at 37°C for 22 hours. Prior to harvesting the biofilms, the optical density (A_590_) of the cultures was determined. The plate was subsequently washed with water, biofilms were heat fixed at 60°C, and the plates and contents were allowed to cool to room temperature. The biofilms were stained with 0.1% crystal violet, washed with water, destained with 33% acetic acid and the absorbance of the resulting solution was recorded at 570 nm and standardized to an acetic acid blank and subsequently to the optical density of the cells upon harvest. Finally the data were normalized with respect to the WT strain to obtain relative biofilm formation.

### Exoprotein analyses and zymography

Spent medium supernatants were obtained from overnight cultures, filter sterilized with a 0.22 μm (pore-size) syringe filters, and standardized to culture optical densities (A_600_). Zymographic analyses of bacteriolytic proteins were conducted using standard methods described elsewhere [98] and samples were separated upon a 12% SDS gel incorporated with 0.3% (vol/vol) heat killed USA300_LAC cells [98]. To determine exoprotein profiles, the spent media supernatant was concentrated using standard trichloroacetic acid precipitation. The resultant protein pellets were resuspended in laemelli buffer and equal volumes were separated upon a 12% SDS gel.

### Bioinformatic analyses

The taxonomic distribution of Suf was determined via BLASTp analyses of publically available genome sequences in October of 2011 as part of a previous study [25]. This distribution of Suf was characterized using the KEGG gene viewer [99], with manual verification using BLASTp or using sequence alignments. 1094 genomes out of a total of 1667 genome sequences (65.6% of total) encoded for SufBC. Genomes that encoded for SufBC were then screened for the presence of SufT using BLASTp. *sufT* was considered to be associated with the *suf* operon if they were within four open reading frames from *sufBC* and appeared to be transcribed from a common promoter.

SufT sequences were compiled and aligned with ClustalW specifying default parameters [100]. The aligned sequences were manually truncated to the minimal SufT sequence or positions 1 to 99 of SufT from *Thermoplasma acidophilum* (Kegg ID: Ta0200). Phyml was used to reconstruct the evolutionary history of the SufT alignment block specifying the Blosum62 substitution model and gamma distributed rate variation [101]. The topology of the tree was evaluated using Chi2-based likelihood ratio tests. The phylogenetic reconstruction was projected with the Interactive Tree Of Life (Itol) web program [102].

The N- and C-terminal sequences that were pruned from the alignment block were subjected to BLASTp against the Conserved Domain Database (CDD) using an evalue of 0.01 [103]. Identified motifs in both N- and C- terminal motifs were compartmentalized into modular structures based on the presence of unique sequence motifs. These N- and C-terminal motifs were mapped onto the SufT phylogenetic tree using the Itol program. Furthermore, SufT was mapped onto a concatenated SufBC phylogenetic tree using the Itol program. The concatenated SufBC tree was constructed as previously described [25].

## Supporting Information

S1 Fig Growth profiles of strains. Panel A: A strain lacking AcnA is nearly incapable of growth in 20AA glutamate medium. Growth traces of the WT (JMB1100) and Δ*acnA* (JMB 1163) strains during aerobic culture are shown. Panel B: Strains lacking SufT or Nfu display growth profiles largely similar to the WT strain in 20AA glucose medium. Growth traces of the WT (JMB1100), Δ*sufT* (JMB1146) and the Δ*nfu* (JMB1165) strains during aerobic culture are shown. Data represent average values of two biological replicates and error bars represent standard deviations.
(TIF)

S2 Fig Transcriptional activity of *acnA* in a strain lacking SufT. The transcriptional activity of the *acnA* gene was assessed in the WT (JMB1100) and the Δ*sufT* (JMB1146) strains carrying a construct containing *gfp* under the transcriptional control of the *acnA* promoter. GFP fluorescence was monitored over time.
(TIF)

S3 Fig Growth profiles upon 18AA glucose medium during micoaerobic culture. Growth traces of the WT (JMB1100), Δ*sufT* (JMB1146) and the Δ*nfu* (JMB1165) strains during microaerobic culture are shown. Data represent average value of four biological replicates and error bars represent standard deviations.
(TIF)

S4 Fig Analyses of superoxide dismutase activity across growth. Sod activity was assessed in cell-free lysates generated from WT (JMB1100) and Δ*sufT* (JMB 1146) strains. The lysates used were the same lysates as used to determine AcnA activity displayed Fig 3B. Data represent the average of three biological replicates and errors bars represent standard deviations.
(TIF)

S5 Fig Superoxide dismutase activity in strains following challenge with paraquat. The *acnA*::*TN* (JMB3537; parent) and the *acnA*::*TN* Δ*sufT* (JMB3539) strains containing p*acnA* were cultured aerobically to post exponential growth phase before one set of cultures was challenged with paraquat for one hour. Sod activity was determined in cell-free lysates. Data represent the average of three biological replicates and errors bars represent standard deviations.
(TIF)

S6 Fig Phenotypic analyses of the Δ*nfu* Δ*sufT* double mutant in complex medium. Panel A, B and C: A Δ*nfu* Δ*sufT* double mutant phenocopies a strain lacking AcnA during aerobic culture in TSB. The WT (JMB1100), Δ*acnA* (JMB 1163), Δ*sufT* (JMB 1146), Δ*nfu* (JMB1165) and the Δ*nfu* Δ*sufT* (JMB2514) strains were cultured aerobically in TSB and the culture optical density (Panel A), pH of the spent media supernatant (Panel B) and acetic acid concentration in the spent media supernatant (Panel C) was assessed periodically. Representative data from one days experiment are displayed.
(TIF)

S7 Fig AcnA activity in strains carrying *sufA* or *sufT* in multicopy. Panel A: AcnA activity is not significantly altered in the Δ*sufT* and Δ*nfu* strains carrying *sufA* upon a multi-copy plasmid. AcnA activity was assessed from the WT (JMB1100), Δ*sufT* (JMB1146), and Δ*nfu* (JMB1165) strains carrying either pEPSA5 (empty vector; pEV) or pEPSA5_*sufA* (p*sufA*). Panel B: AcnA activity is not significantly altered in the Δ*nfu* strain carrying *sufT* upon a multi-copy plasmid. AcnA activity was assessed from the WT (JMB1100) and Δ*nfu* (JMB1165) strains carrying either pCM28 (empty vector; pEV) or pCM28_*sufT* (p*sufT*).
(TIF)

S8 Fig Assessing the growth of a Δ*sufT* mutant carrying *nfu* in multicopy in 20AA glutamate medium. Growth traces are displayed for the WT (JMB1100) and Δ*sufT* (JMB1146) strains carrying either pEPSA5 (empty vector) or pEPSA5_*nfu*. Strains were cultured aerobically in 20AA glutamate media in the absence (Panel A) or presence (Panel B) of xylose to induce *nfu* transcription. Data represent the average of two biological replicates and standard deviations are shown.
(TIF)

S9 Fig Growth in the presence of vancomycin. Growth inhibition in the presence of varying concentrations of vancomycin was assessed in WT (JMB1100) and the Δ*nfu* Δ*sufT* (JMB2514) strains.
(TIF)

S1 Table Oligonucleotides used in this study. (DOCX)
