# Supplementary figures and images for: The DUF59 Containing Protein SufT Is Involved in the Maturation of Iron-Sulfur (FeS) Proteins during Conditions of High FeS Cofactor Demand in Staphylococcus aureus
Source: PLoS Genet. 2016 Aug 12;12(8):e1006233. doi: 10.1371/journal.pgen.1006233 (PMC4982691; doi:10.1371/journal.pgen.1006233)

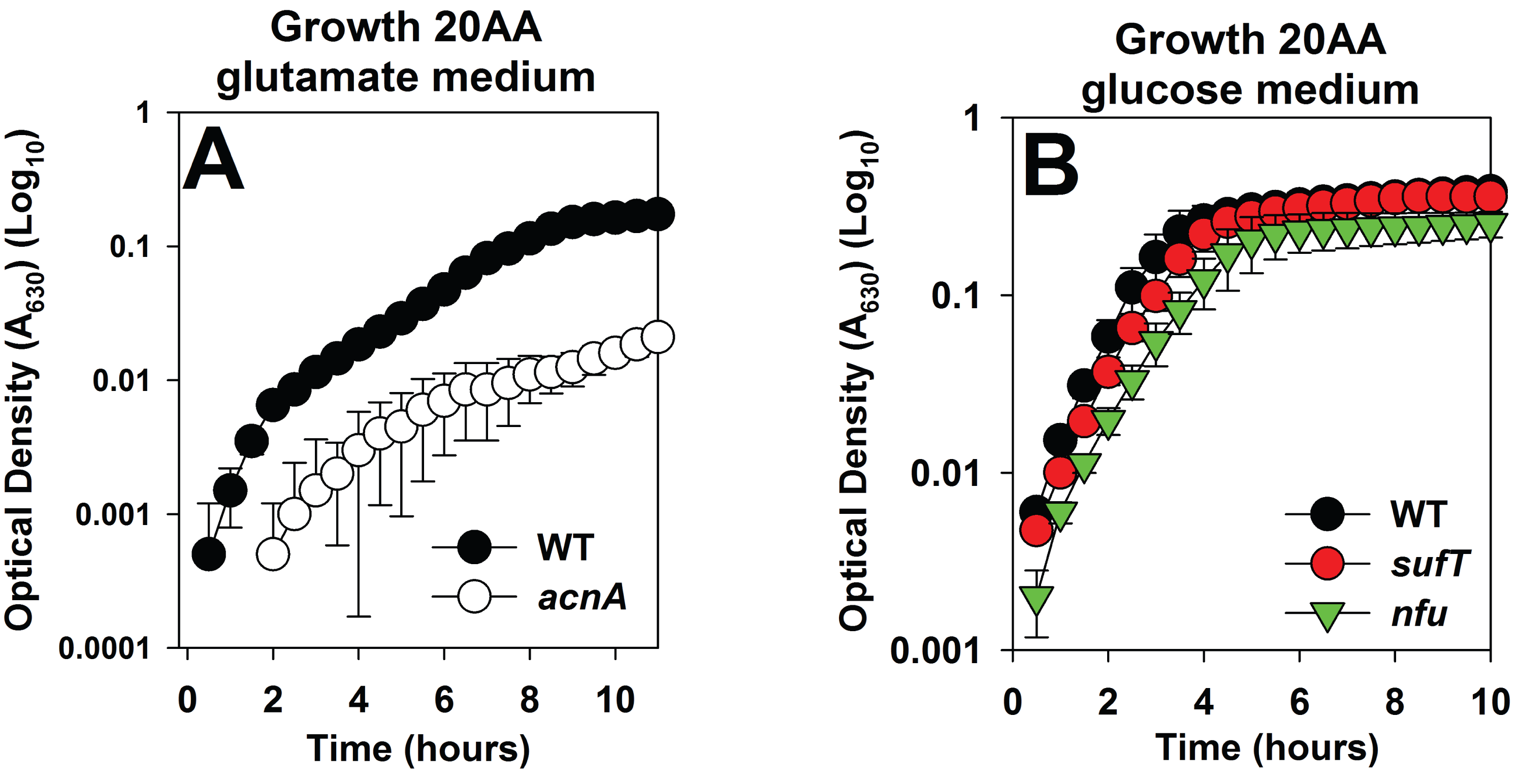

Supplement: S1 Fig — Panel A: A strain lacking AcnA is nearly incapable of growth in 20AA glutamate medium. Growth traces of the WT (JMB1100) and ΔacnA (JMB 1163) strains during aerobic culture are shown. Panel B: Strains lacking SufT or Nfu display growth profiles largely similar to the WT strain in 20AA glucose medium. Growth traces of the WT (JMB1100), ΔsufT (JMB1146) and the Δnfu (JMB1165) strains during aerobic culture are shown. Data represent average values of two biological replicates and error bars represent standard deviations. (TIF) [file pgen.1006233.s001.tif]

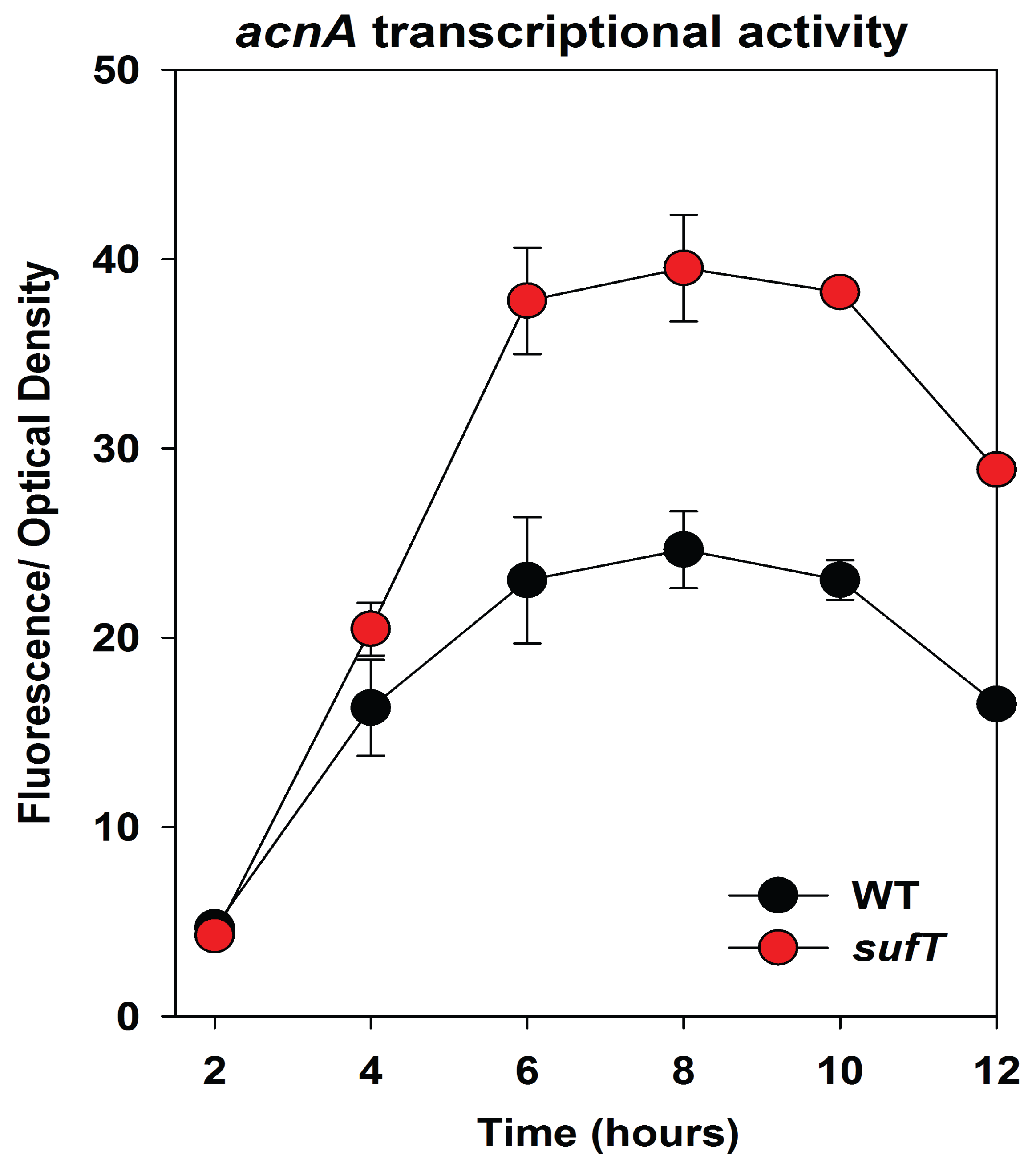

Supplement: S2 Fig — The transcriptional activity of the acnA gene was assessed in the WT (JMB1100) and the ΔsufT (JMB1146) strains carrying a construct containing gfp under the transcriptional control of the acnA promoter. GFP fluorescence was monitored over time. (TIF) [file pgen.1006233.s002.tif]

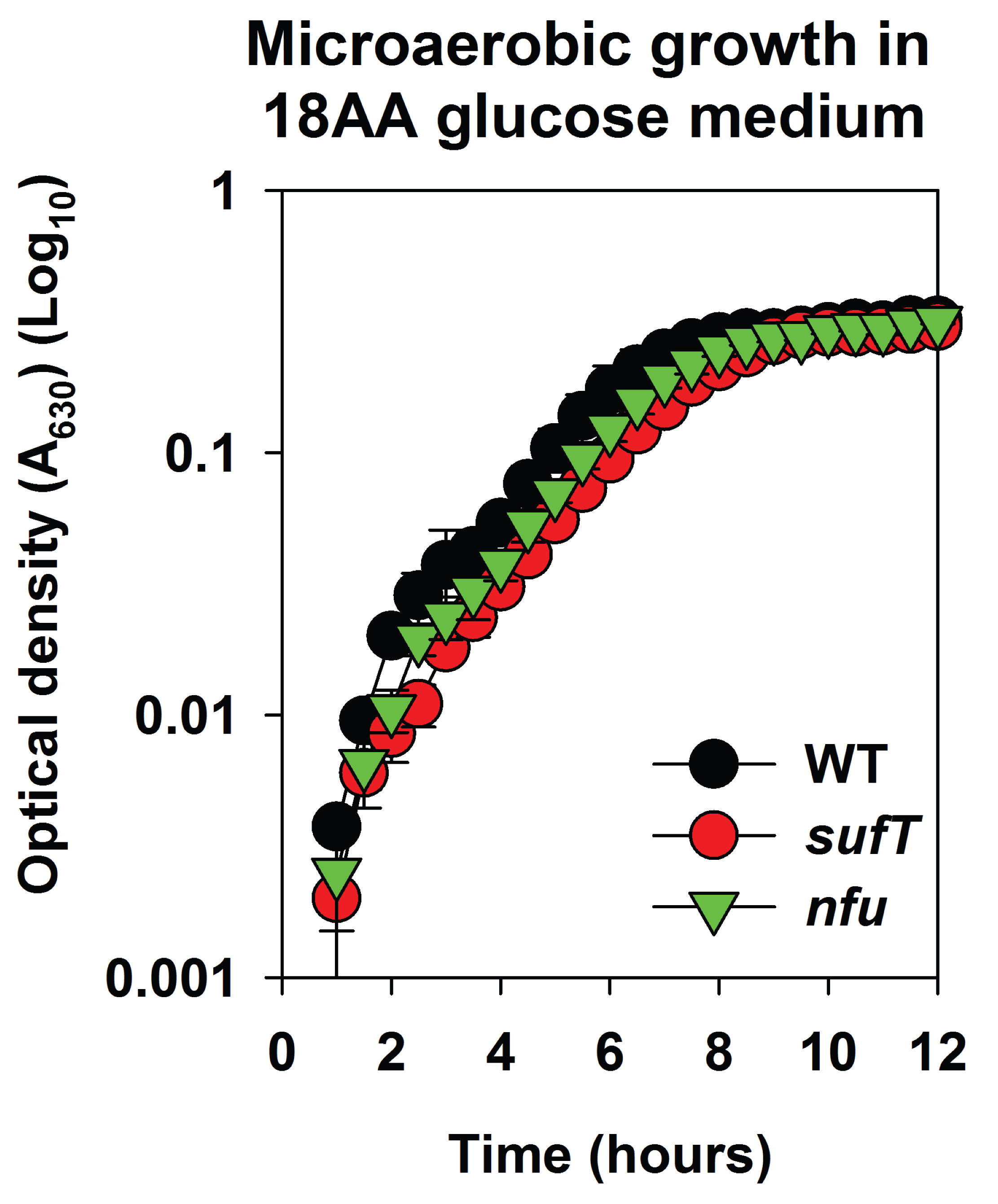

Supplement: S3 Fig — Growth traces of the WT (JMB1100), ΔsufT (JMB1146) and the Δnfu (JMB1165) strains during microaerobic culture are shown. Data represent average value of four biological replicates and error bars represent standard deviations. (TIF) [file pgen.1006233.s003.tif]

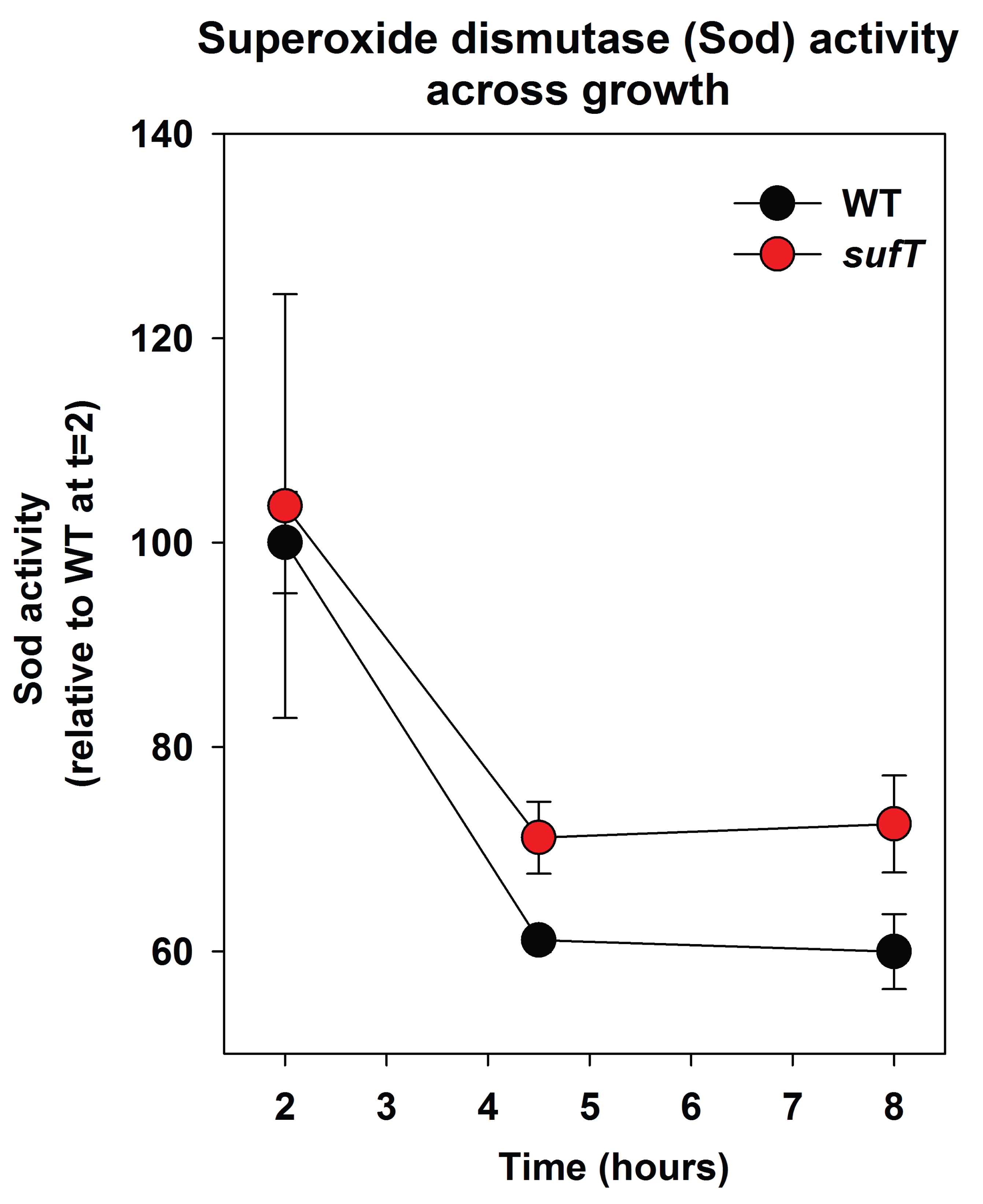

Supplement: S4 Fig — Sod activity was assessed in cell-free lysates generated from WT (JMB1100) and ΔsufT (JMB 1146) strains. The lysates used were the same lysates as used to determine AcnA activity displayed Fig 3B. Data represent the average of three biological replicates and errors bars represent standard deviations. (TIF) [file pgen.1006233.s004.tif]

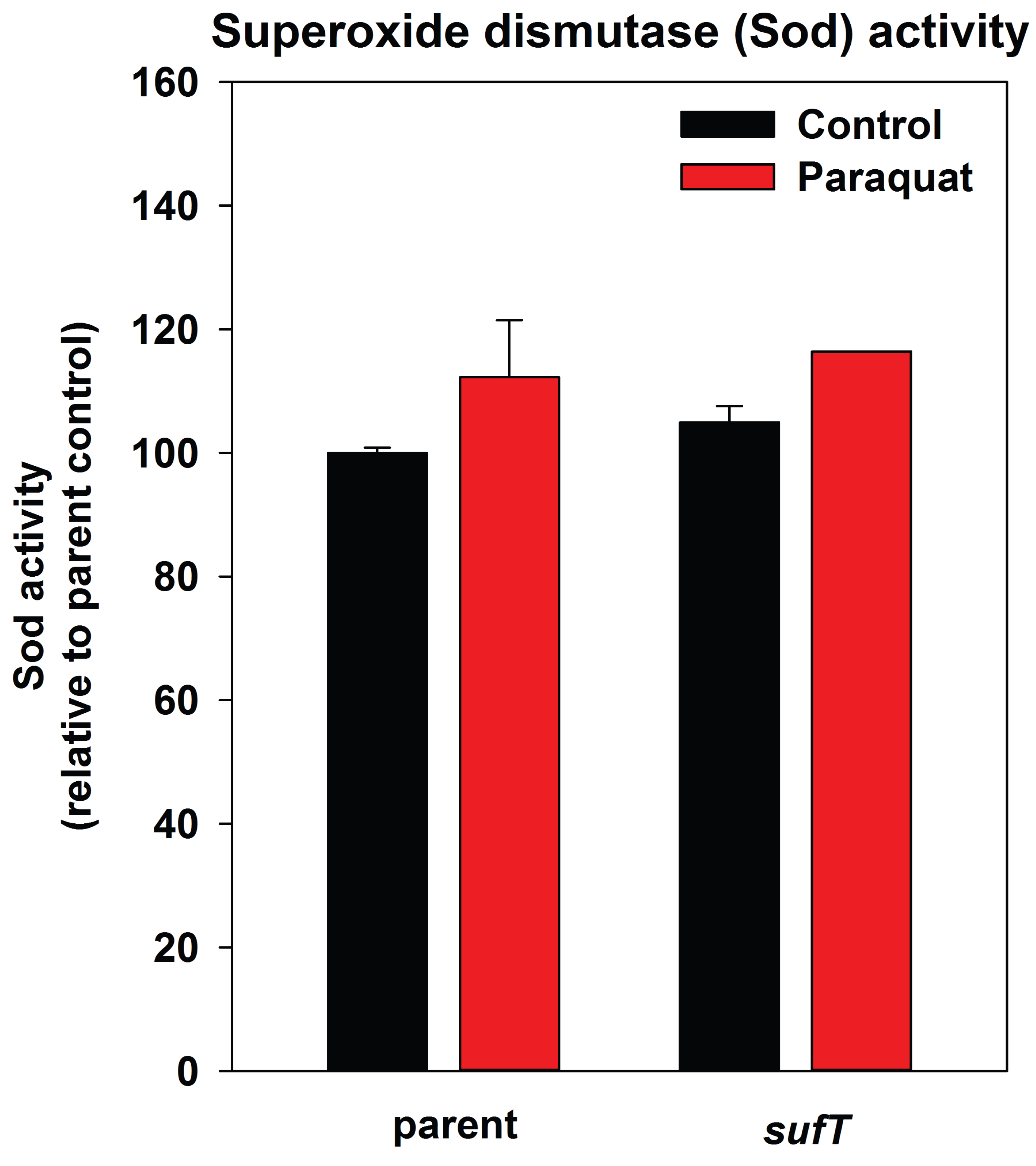

Supplement: S5 Fig — The acnA::TN (JMB3537; parent) and the acnA::TN ΔsufT (JMB3539) strains containing pacnA were cultured aerobically to post exponential growth phase before one set of cultures was challenged with paraquat for one hour. Sod activity was determined in cell-free lysates. Data represent the average of three biological replicates and errors bars represent standard deviations. (TIF) [file pgen.1006233.s005.tif]

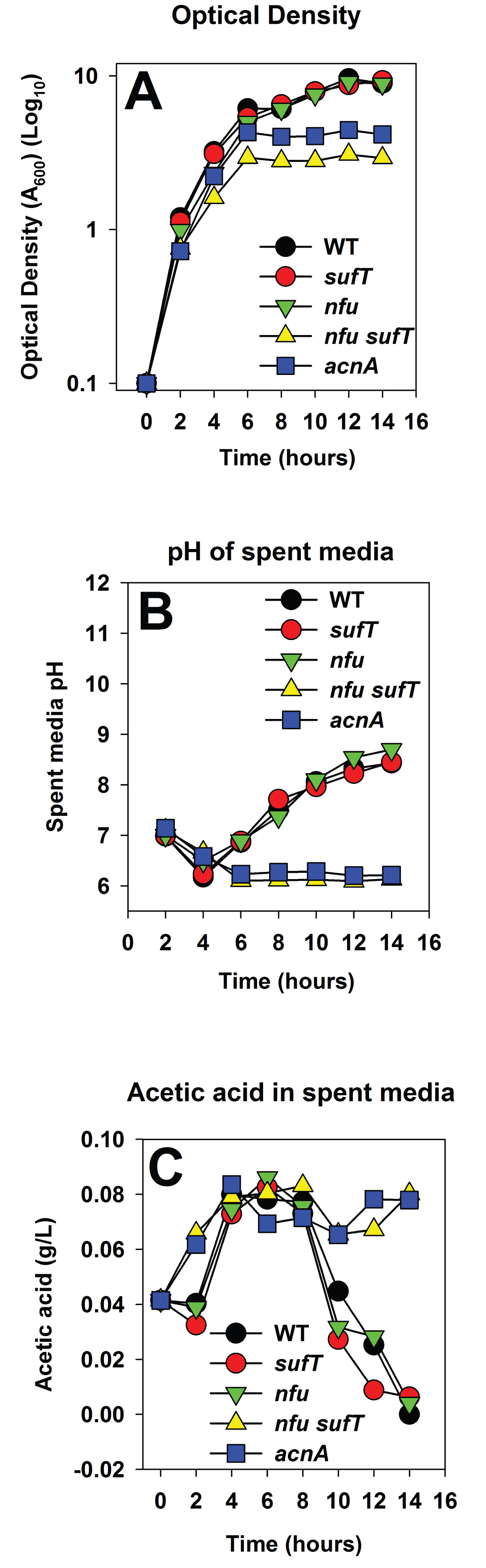

Supplement: S6 Fig — Panel A, B and C: A Δnfu ΔsufT double mutant phenocopies a strain lacking AcnA during aerobic culture in TSB. The WT (JMB1100), ΔacnA (JMB 1163), ΔsufT (JMB 1146), Δnfu (JMB1165) and the Δnfu ΔsufT (JMB2514) strains were cultured aerobically in TSB and the culture optical density (Panel A), pH of the spent media supernatant (Panel B) and acetic acid concentration in the spent media supernatant (Panel C) was assessed periodically. Representative data from one days experiment are displayed. (TIF) [file pgen.1006233.s006.tif]

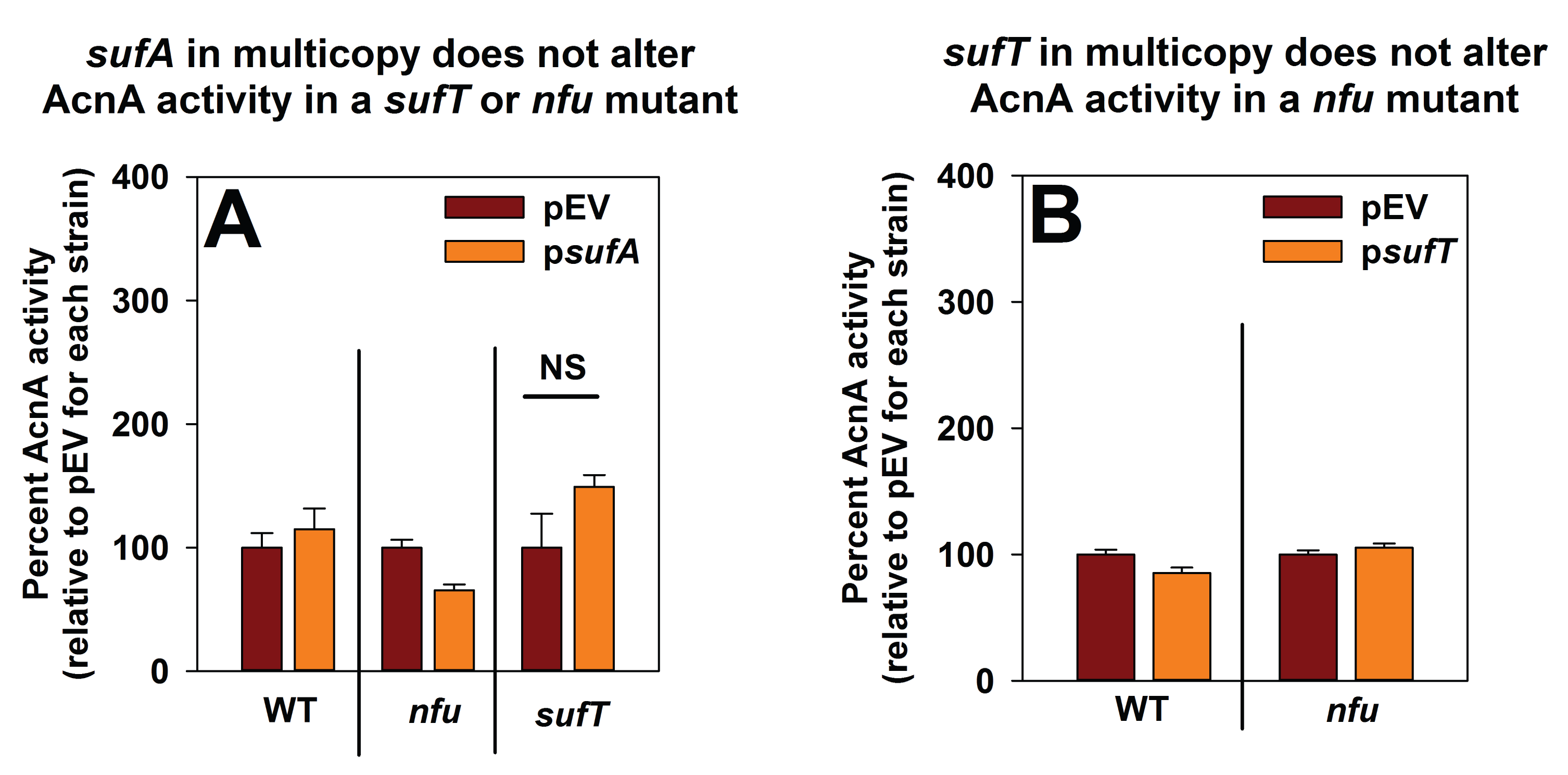

Supplement: S7 Fig — Panel A: AcnA activity is not significantly altered in the ΔsufT and Δnfu strains carrying sufA upon a multi-copy plasmid. AcnA activity was assessed from the WT (JMB1100), ΔsufT (JMB1146), and Δnfu (JMB1165) strains carrying either pEPSA5 (empty vector; pEV) or pEPSA5_sufA (psufA). Panel B: AcnA activity is not significantly altered in the Δnfu strain carrying sufT upon a multi-copy plasmid. AcnA activity was assessed from the WT (JMB1100) and Δnfu (JMB1165) strains carrying either pCM28 (empty vector; pEV) or pCM28_sufT (psufT). (TIF) [file pgen.1006233.s007.tif]

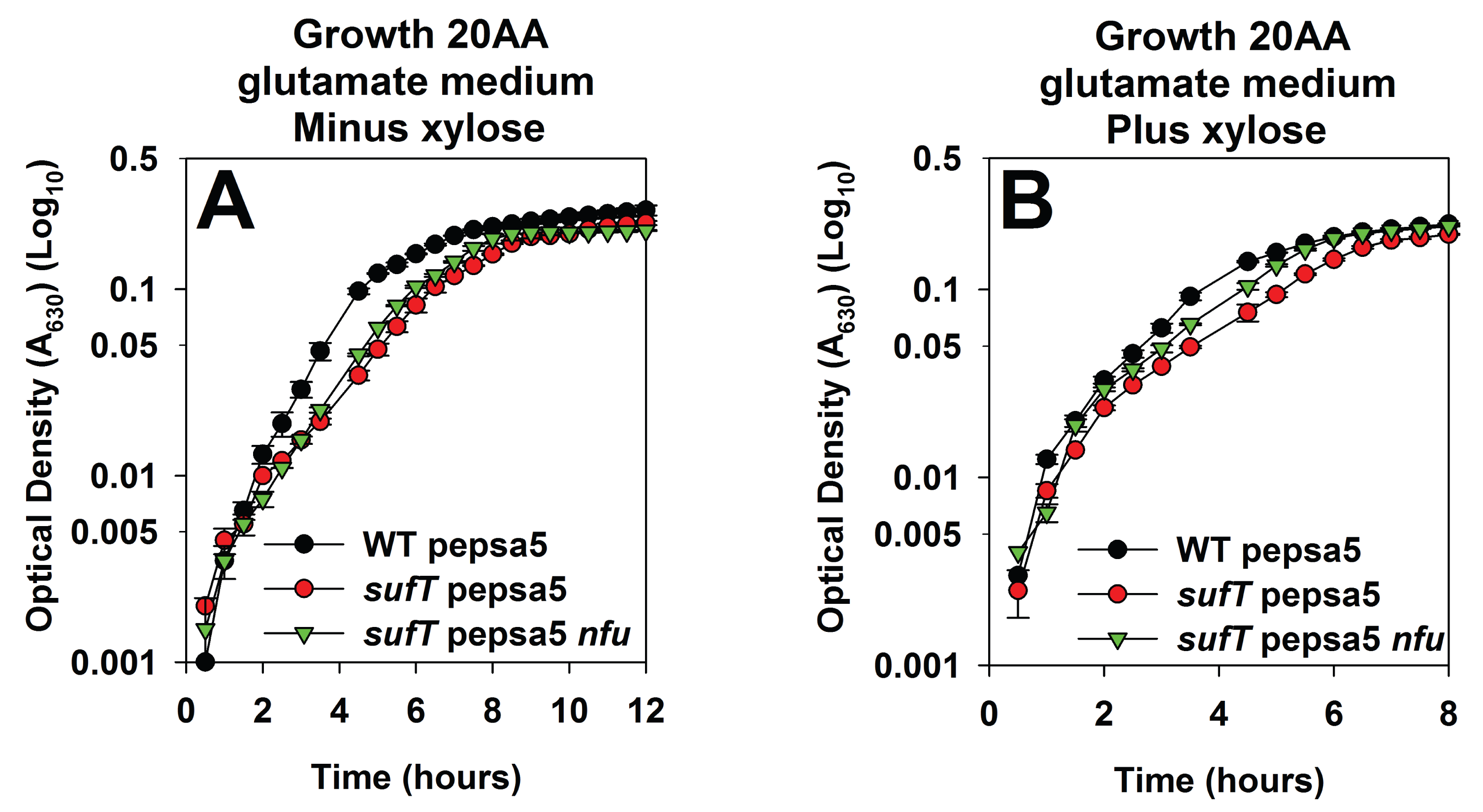

Supplement: S8 Fig — Growth traces are displayed for the WT (JMB1100) and ΔsufT (JMB1146) strains carrying either pEPSA5 (empty vector) or pEPSA5_nfu. Strains were cultured aerobically in 20AA glutamate media in the absence (Panel A) or presence (Panel B) of xylose to induce nfu transcription. Data represent the average of two biological replicates and standard deviations are shown. (TIF) [file pgen.1006233.s008.tif]

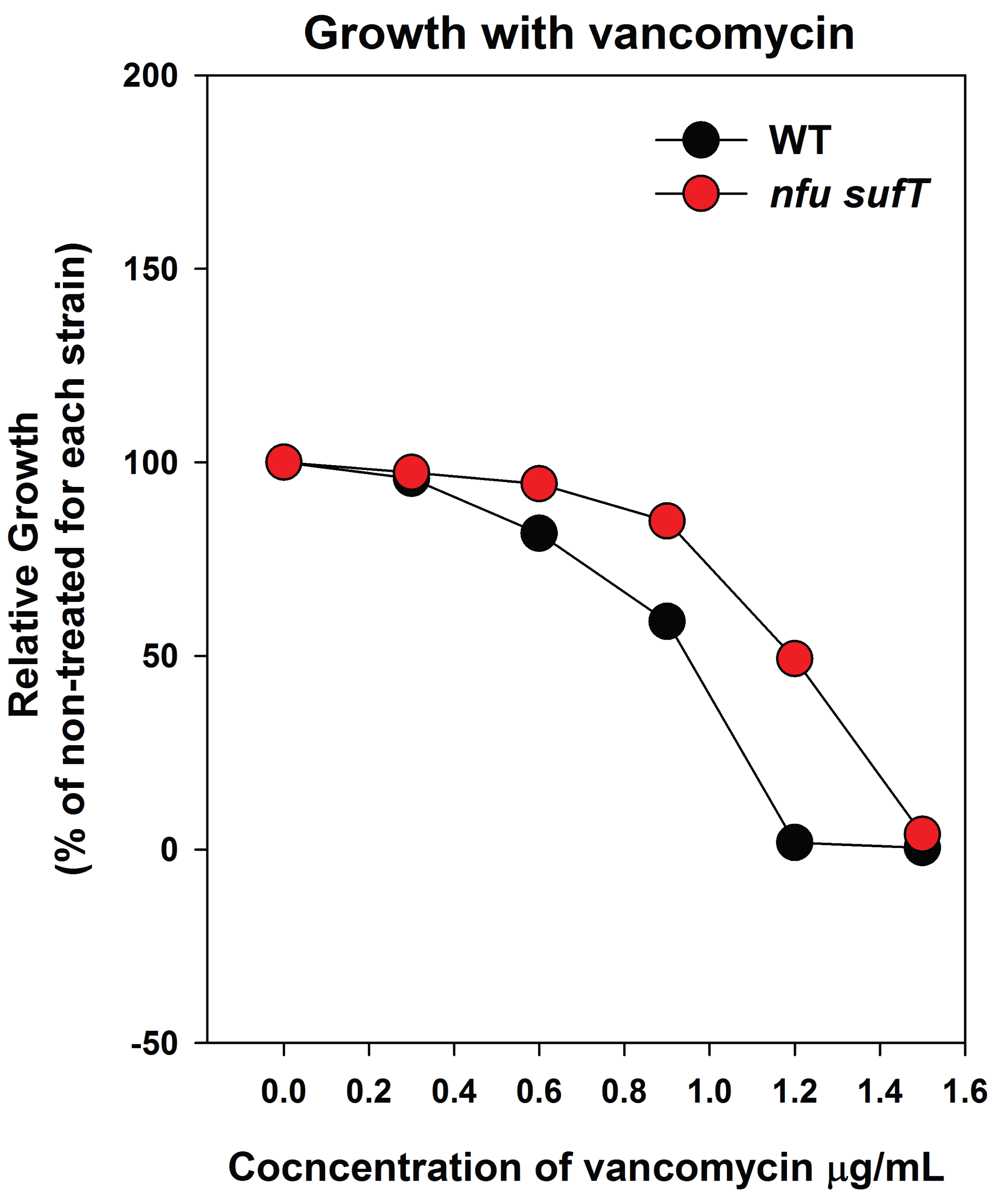

Supplement: S9 Fig — Growth inhibition in the presence of varying concentrations of vancomycin was assessed in WT (JMB1100) and the Δnfu ΔsufT (JMB2514) strains. (TIF) [file pgen.1006233.s009.tif]
