# Supplementary material for: The DUF59 Containing Protein SufT Is Involved in the Maturation of Iron-Sulfur (FeS) Proteins during Conditions of High FeS Cofactor Demand in Staphylococcus aureus
Source: PLoS Genet. 2016 Aug 12;12(8):e1006233. doi: 10.1371/journal.pgen.1006233 (PMC4982691; doi:10.1371/journal.pgen.1006233)
Supplement: S1 Table — (DOCX) [file pgen.1006233.s010.docx]

| **Table S1: Oligonucleotides used in this study** | |
| --- | --- |
| **RT- PCR primers** | |
| sufT5RT | AATGTTGATGATGAAGGCGTATGT |
| sufT3RT | CAATAATTTGAGGTCCCATTGGA |
| sufCRT5 | GATGAAATCGATTCAGGGTTAGACA |
| sufCRT3 | TTCCCCACGCATTTGGTTA |
|  |  |
| **Transcriptional reporter primers** | |
| 875gfpKpnI | gggggtaccGCACCTAAGATACTATCTTTCAATGCCTC |
| 875gfpHindIII | gggaagcttCGATACTTGATATTGTTGTAATCCTTG |
| AcnApHindIII | gggaagcttCTGACATTCTTGAAAAGCGTCAGTCACGTAG |
| AcnApKpnI | gggggtaccCCCCCTTGATACATTTTTATATTTATATGCC |
| Sufgfp5hindIII  Sufgfp3kpnI  nfuprokpn5  nfuprohind3 | GGGAAGCTTCTCGTTCCCATAGCAAAACCTTTTAG  GGGCCATGGCCGTAAAATATAAAGTTTTCTTAACTAG  GGGggtaccGTTGTCATTATCTTTTGTAATATCTATATACG  GGGaacgttCGAGAGATGAATTTGATAATCTTTATTAG |
|  |  |
| **Cloning primers** |  |
| 0875up5EcoRI | GGGGAATTCCTTCTGCTATAATATTCGATACTTGATATTGTTG |
| 0875up3NheI | ACGCGTGGTACCGCTAGCGCTAGCGCACCTAAGATACTATCTTTCAATGCCTCTTCC |
| 0875dwn5MluI | GCTAGCGGTACCACGCGTACGCGTGGGCCCGCAGTCAATATAACATTGTAGAGTATA |
| 0875 dwn3BamHI | CCCGGATCCCGAAATCCAATTTGTAATGCAACTCGGCAAC |
| 0875_5BamHI | CCCGGATCCGCGCCATTAAAATTAACAATTGGTGTCGTTAAATTT |
| 0875_3SalI | CCCGTCGACGCGGGCCCAAACACAGAGAATTTCGAA |
| nfuveri5 | GGCGCATTAACGCAACTTGCACATA |
| nfuveri3 | GGATGAGCAAATTGCGAAACATATGAAAGG |
| G+tetnheI | CCCgctagcCGGATTTTATGACCGATGATGAAG |
| G+tetmluI | CCCacgcgtTTAGAAATCCCTTTGAGAATGTTT |
| MT875trunk5 | AGATAGTATCTTAGGTGCATTAGAAATGGTTGAAGAAGCAATGCGAGAC |
| MT875trunk3 | CGTCTCGCATTGCTTCTTCAACCATTTCTAATGCACCTAAGATACTATCT |
| pCM28YCC | AAACCTACAGAAGCTTGCATGCCTGCAGGTCGACGGTGGCACTTTTCGGGGAAAAGTTA |
| ycc875p5 | TAATATAGCGTAACTATAACGGTCCTAAACGCGTGCGCCATTAAAATTAACAATT |
| YCC875P3 | CACCAATTGTTAATTTTAATGGCGCACGCGTTTAGGACCGTTATAGTTACGCTATATTA |
| 875P875G5 | AGATAGTATCTTAGGTGCATTAGAAATGGTAATTGACCCTGAATTAGGAA |
| 875P875G3  875PCM28 3 | TTCCTAATTCAGGGTCAATTACCATTTCTAATGCACCTAAGATACTATCT  GATTACGAATTCATGATCGAATGCTAGCGGATCCGAGCTCGCGGGCCCAAACACAGAGA |
| 875PMT5 | AGATAGTATCTTAGGTGCATTAGAAATGAGTGAAACAAGTGCGCCTGCTGAAG |
| 875PMT3 | CTTCAGCAGGCGCACTTGTTTCACTCATTTCTAATGCACCTAAGATACTATCT |
|  |  |
